# Control of a chemical chaperone by a universally conserved ATPase

**DOI:** 10.1016/j.isci.2024.110215

**Published:** 2024-06-08

**Authors:** Hong Jiang, Martin Milanov, Gabriela Jüngert, Larissa Angebauer, Clara Flender, Eva Smudde, Fabian Gather, Tanja Vogel, Henning J. Jessen, Hans-Georg Koch

**Affiliations:** 1 Institute of Biochemistry and Molecular Biology, ZBMZ, Faculty of Medicine, Albert-Ludwigs-University Freiburg, 79104 Freiburg, Germany; 2 Faculty of Biology, Albert-Ludwigs-University Freiburg, 79104 Freiburg, Germany; 3 Spemann Graduate School of Biology and Medicine, Albert-Ludwigs University Freiburg, 79104 Freiburg, Germany; 4 Institute for Anatomy and Cell Biology, Department of Molecular Embryology, Faculty of Medicine, Albert-Ludwigs-University Freiburg, 79104 Freiburg, Germany; 5 Institute for Organic Chemistry, Faculty of Chemistry and Pharmacy, University Freiburg 79104 Freiburg, Germany

**Keywords:** Applied sciences, Biotechnology, Medical biochemistry

## Abstract

The universally conserved YchF/Ola1 ATPases regulate stress response pathways in prokaryotes and eukaryotes. Deletion of YchF/Ola1 leads to increased resistance against environmental stressors, such as reactive oxygen species, while their upregulation is associated with tumorigenesis in humans. The current study shows that in *E. coli*, the absence of YchF stimulates the synthesis of the alternative sigma factor RpoS by a transcription-independent mechanism. Elevated levels of RpoS then enhance the transcription of major stress-responsive genes. In addition, the deletion of *ychF* increases the levels of polyphosphate kinase, which in turn boosts the production of the evolutionary conserved and ancient chemical chaperone polyphosphate. This potentially provides a unifying concept for the increased stress resistance in bacteria and eukaryotes upon YchF/Ola1 deletion. Intriguingly, the simultaneous deletion of *ychF* and the polyphosphate-degrading enzyme exopolyphosphatase causes synthetic lethality in *E. coli*, demonstrating that polyphosphate production needs to be fine-tuned to prevent toxicity.

## Highlights

• The universally conserved ATPase YchF inhibits synthesis of RpoS and polyphosphate kinase (PPK)
• Upon depletion of YchF, the levels of the chemical chaperone polyphosphate increase
• Increased RpoS and polyphosphate levels explain stress resistance of Δ*ychF* cells

## Graphical abstract

**Figure:**
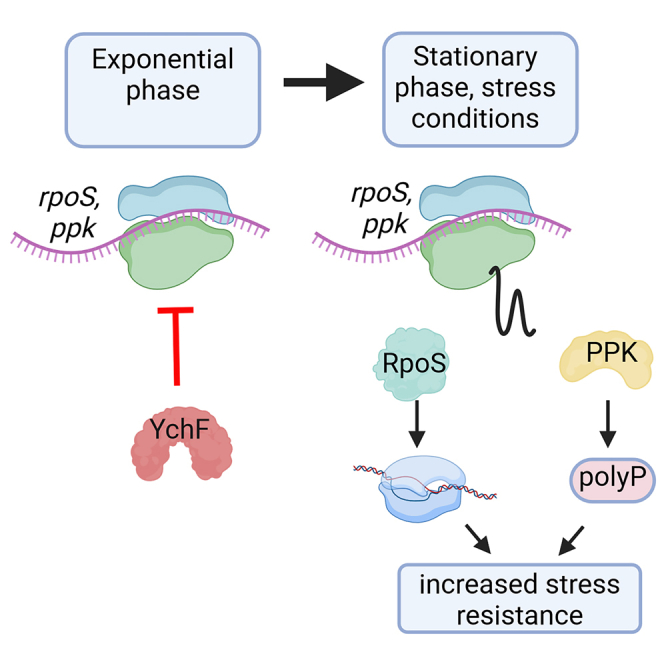

## Introduction

The cellular stress response comprises a wide range of molecular changes that cells undergo in response to intracellular or extracellular stressors, such as temperature or pH variations, nutrient limitation, and exposure to toxins or mechanical damage.^1^^,^^2^^,^^3^^,^^4^ The stress response is also triggered when cells are attacked by pathogens^5^ or face intense competition in their natural environments.^6^^,^^7^ Unicellular organisms, such as bacteria, are particularly exposed to environmental challenges and therefore execute a wide range of stress response mechanisms, which often act in parallel and control the expression of a large number of stress response proteins.^8^^,^^9^^,^^10^^,^^11^ Bacteria regulate gene expression primarily at the level of transcription by modulating promoter binding and promoter specificity of RNA polymerase (RNAP). This involves stress-induced sigma factors, which bind as regulatory subunits to the RNAP-core and confer promoter specificity,^12^ or transcription factors, such as DksA, which binds to RNAP and regulates transcription.^13^^,^^14^ Transcription can be further controlled by small RNAs,^15^^,^^16^ which also regulate translation.^17^^,^^18^ The multi-faceted bacterial stress response furthermore includes small signaling molecules, such as the nucleotide derivatives (p)ppGpp, which like sigma factors define promoter specificity of RNAP^19^^,^^20^ and act as competitive inhibitors of GTP-dependent proteins.^4^^,^^21^^,^^22^ The control of stress response pathways by sigma factors is typical for bacteria and not observed in archaea or eukaryotes.^12^ Likewise, (p)ppGpp is mainly found in bacteria and bacteria-derived chloroplasts, with only a few reports on (p)ppGpp in metazoa.^23^ Still, there are also universally conserved regulators of stress response pathways and one example is the ATPase YchF and its eukaryotic homolog Ola1.^24^^,^^25^^,^^26^^,^^27^^,^^28^ YchF/Ola1 are members of the translation-factor-related superfamily of GTPases,^29^ but they preferentially hydrolyze ATP rather than GTP due to a small variation within the nucleotide-binding site.^25^ In bacteria, yeast, plants, and mammals, YchF/Ola1 has been linked to multiple stress response mechanisms^27^^,^^28^^,^^30^^,^^31^^,^^32^^,^^33^^,^^34^^,^^35^^,^^36^ and its overproduction is observed in multiple types of cancer and potentially associated with poor survival rates in human cancer patients.^37^^,^^38^ When YchF/Ola1 is absent or depleted in *E. coli*, or plant and mammalian cells, cells show increased stress resistance,^27^^,^^28^^,^^33^^,^^39^ suggesting that YchF/Ola1 acts as a negative regulator of stress response pathways. This is potentially linked to its ability to bind to ribosomes^31^^,^^40^^,^^41^^,^^42^^,^^43^ and to suppress the translation of stress-responsive mRNAs.^3^^,^^30^^,^^41^ In contrast, yeast Ola1 acts as a positive regulator of the heat shock response^31^ and it was shown to stabilize Hsp70 in mammalian cells.^32^ Thus, the YchF/Ola1-dependent regulation of stress response pathways likely involves both ribosome-dependent and -independent mechanisms.^3^^,^^24^^,^^44^

In the model organism *E. coli*, YchF interacts with anti-oxidative proteins, such as catalases or glutaredoxins,^27^^,^^39^ but biochemical assays did not show any indication for a direct inhibition of catalases by YchF.^41^ In addition, the absence of YchF in *E. coli* not only increases resistance against oxidative stress but also the resistance against replication and translation stress,^27^^,^^39^^,^^41^ suggesting that YchF controls superordinate regulatory mechanisms.

In the current study, the consequences of *ychF* deletion in *E. coli* were analyzed and the data show that the absence of YchF induces the RpoS response, which acts as the major stress response pathway in bacteria,^9^ and leads to an upregulation of polyphosphate (polyP) synthesis, which serves as an ancient and universally conserved chemical chaperone.

## Results

### The Δ*ychF* strain shows increased RpoS levels

**Figure 1:**
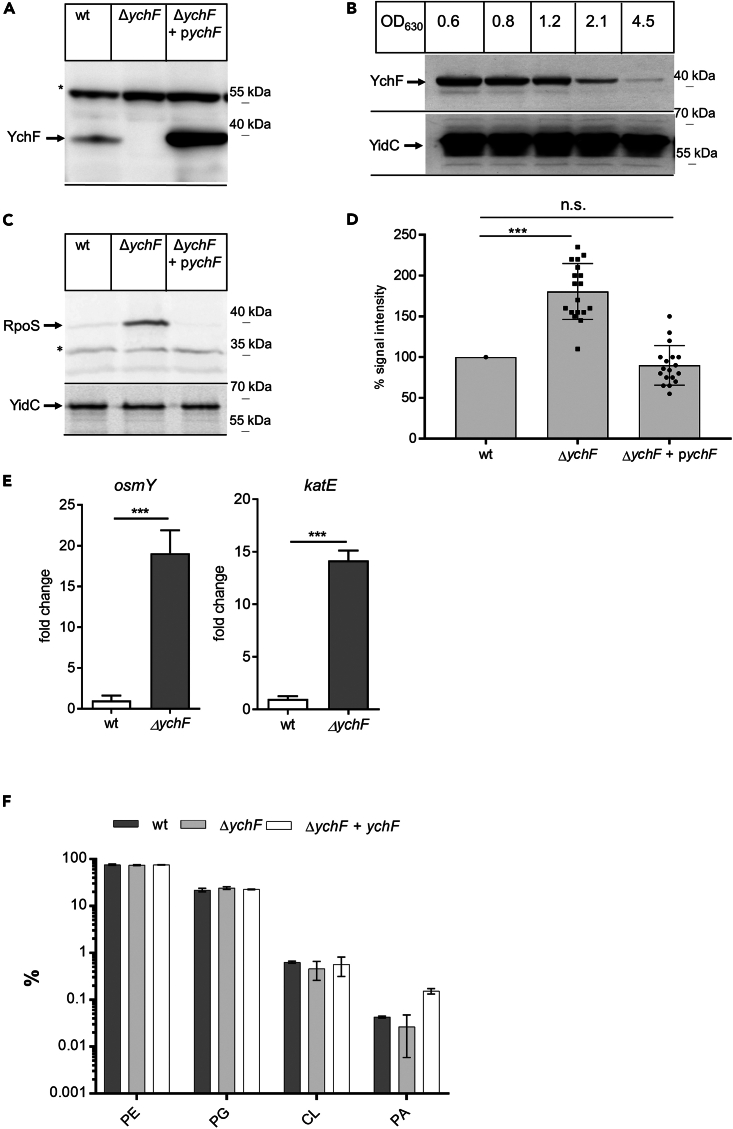
The absence of YchF increases the RpoS levels in *E. coli* (A) Wild type (wt), the Δ*ychF* strain, and the Δ*ychF* strain containing *ychF* on a plasmid under the arabinose promoter were grown on LB medium or LB medium supplemented with 0.01% arabinose for Δ*ychF* + p*ychF* up to an optical density (OD_600_) of 1.5 and cells were then precipitated with trichloroacetic acid (TCA) and separated by SDS-PAGE, followed by western blotting and immune detection using α-YchF antibodies. ∗ indicates a band that is unspecifically recognized by α-YchF antibodies. A representative blot of *n* ≥ 3 experiments is shown. (B) Growth-phase-dependent production of YchF. *E. coli* wt cells were grown on LB medium up to the indicated OD and processed as in (A). The membrane after blotting was cut into two pieces, which were either decorated with α-YchF antibodies or with α-YidC antibodies as loading control. A representative blot of *n* ≥ 3 experiments is shown. (C) The *E. coli* strains were grown on LB medium up to OD_6__00_ 0.8 and processed as in (A). One part of the membrane was incubated with α-RpoS antibodies, and the other part with α-YidC antibodies. ∗ indicates a band that is unspecifically recognized by α-RpoS antibodies. See also Figure S1. (D) The α-RpoS western blot signals of several independent experiments as shown in Figure 1C (*n* = 18; cells grown on LB medium to OD_600_ ∼0.8) were quantified by ImageJ. Signal intensities of wt cells in individual experiments were set to 100% and the intensities in the Δ*ychF* and Δ*ychF* + p*ychF* strains were calculated and visualized by GraphPad prism. The error bars reflect the standard deviation. To determine the significance of the results, the *p* value was calculated using the “unpaired, two-tailed t test” of the program GraphPad prism. The *p* values are depicted as asterisks (∗) above the graphs as following: not significant (n.s.) = *p* > 0.05; ∗∗∗ = *p* ≤ 0.001. (E) RT-qPCR of wt and Δ*ychF* cells, grown in LB medium until OD_600_ = 0.8. Total cellular RNAs were extracted from 10^8^ cells, using the RNAeasy mini Kit (QIAGEN) and samples were treated with DNaseI. Using random primers, a cellular cDNA library was created of 1 μg total RNA by reverse transcription. The cDNA amount of the target genes *katE*, *osmY* and the reference gene *hcaT* were analyzed by qPCR, as described in Material and Methods. The graph shows the mean values of the fold change of the expression of *katE* and *osmY* in biological triplicates from Δ*ychF* cells (gray) compared to wt cells (white). The error bars reflect the standard deviation. To determine the significance of the results, the *p* value was calculated using the “unpaired, two-tailed t test” of the program GraphPad Prism 6. The *p* values are depicted as asterisks (∗) above the graphs as following: n.s. = *p* > 0.05; ∗ = *p* ≤ 0.05; ∗∗ = *p* ≤ 0.01; ∗∗∗ = *p* ≤ 0.001. (F) Lipidomic analyses of isolated inner membrane vesicles (INVs) derived from the indicated strains. The extraction, analysis, and quantification were performed by Lipotype GmbH, Dresden, Germany, as described in Material and Methods. Shown are the percentages of the main *E. coli* lipids (phosphatidylethanolamine, PE; phosphatidylglycerol, PG; cardiolipin, CL; and phosphatidic acid, PA). The error bars reflect the standard deviation (*n* = 3). The “unpaired, two-tailed t test” of the program GraphPad prism revealed no significant differences in the lipid composition between wt, Δ*ychF*, and Δ*ychF* + p*ychF* strains (*p* > 0.05). For the sake of clarity, the *p* values are not displayed in the figure.

A major regulator of the bacterial stress response is the sigma factor RpoS, which enhances the transcription of stress-responsive genes by RNAP.^45^^,^^46^^,^^47^ The RpoS regulon includes approx. 500 genes in *E. coli*^11^ and is primarily activated when cells encounter nutrient starvation or enter the stationary growth phase.^47^ The cellular RpoS levels are controlled by a complex regulatory network that includes transcription, translation, and degradation.^9^ In addition, the alarmone (p)ppGpp regulates the ability of RpoS to compete with the housekeeping sigma factor RpoD.^48^ Whether YchF influences the RpoS levels was analyzed by monitoring the RpoS levels in *E. coli* wild-type cells, the Δ*ychF* and the Δ*ychF* + p*ychF* strains; the latter contains *ychF* on a plasmid under the control of the arabinose promoter (Figure 1A). In wild-type *E. coli* cells, YchF expression is growth phase dependent and expression is downregulated when cells enter stationary phase (Figure 1B) or encounter stress conditions.^41^ When the RpoS levels were monitored in exponentially grown cells, we noticed that the RpoS levels in the Δ*ychF* cells were significantly increased compared to wild-type cells or Δ*ychF* cells containing the plasmid-encoded *ychF* copy (Figure 1C). The quantification of several independent experiments (*n* = 18) verified that the RpoS levels in *E. coli* are inversely correlated to the YchF levels (Figure 1D). The RpoS levels in these strains were also monitored at different optical densities (Figure S1), which revealed that the Δ*ychF* strain had already increased RpoS levels during early exponential phase, which did not drastically change with increasing optical densities (ODs) (Figure S1). In contrast, in wild-type cells, the RpoS levels increased with increasing ODs and reached similar levels as in the Δ*ychF* strain only beyond OD 2.1 (Figure S1). In the Δ*ychF* + p*ychF* strain, the RpoS levels were generally low and were almost undetectable even at OD 4.5 (Figure S1). These data indicate that the absence of YchF stimulates the production of the general stress-responsive sigma factor RpoS and that the increased RpoS levels in stationary wild-type cells coincide with the reduced YchF levels. In contrast, elevated levels of YchF reduce the RpoS levels in *E. coli* cells.

For validating that the absence of YchF induces the RpoS response, we determined the mRNA levels of two RpoS-controlled genes, *osmY* and *katE* by quantitative reverse-transcription PCR (qRT-PCR), using the *hcaT* mRNA as a housekeeping gene. *hcaT* encodes for a putative 3-phenylpropionate transporter and was identified as a very stable reference transcript for qPCR.^49^ OsmY is a periplasmic chaperone that is induced by hyperosmotic stress,^50^ and KatE is the major catalase during stationary phase and also induced by hyperosmotic stress.^11^^,^^51^ For both genes, we observed a significant increase in their respective mRNA levels (Figure 1E), verifying that the increased RpoS levels in the Δ*ychF* strain induce the general stress response. The upregulation of KatE also explains the enhanced catalase activity that is observed in cell extracts of the Δ*ychF* strain.^27^

Although RpoS is important for the transition of *E. coli* cells into stationary phase, some metabolic changes in stationary phase are independent of RpoS. This is exemplified by the cardiolipin content in the membrane, which increases during stationary phase.^52^ *E. coli* contains three cardiolipin synthases, ClsA, ClsB, and ClsC,^53^^,^^54^ yet their expression is not controlled by RpoS, but instead by osmolarity in case of ClsA,^55^ by the alarmone ppGpp in case of ClsB,^56^ and by cold shock in case of ClsC.^57^ Monitoring the cardiolipin content in the membrane therefore reveals whether the absence of *ychF* activates multiple stress response pathways in addition to the RpoS response. The lipid composition of the *E. coli* inner membrane was determined by a lipidomics approach, which however did not reveal major differences in the amounts of phosphatidylethanolamine, phosphatidylglycerol, cardiolipin, or phosphatidic acid between wild-type, the Δ*ychF*, and the complemented Δ*ychF* strain (Figure 1F). Thus, the absence of YchF does not induce all stationary phase-associated stress response pathways.

### The absence of YchF favors *rpoS* translation

**Figure 2:**
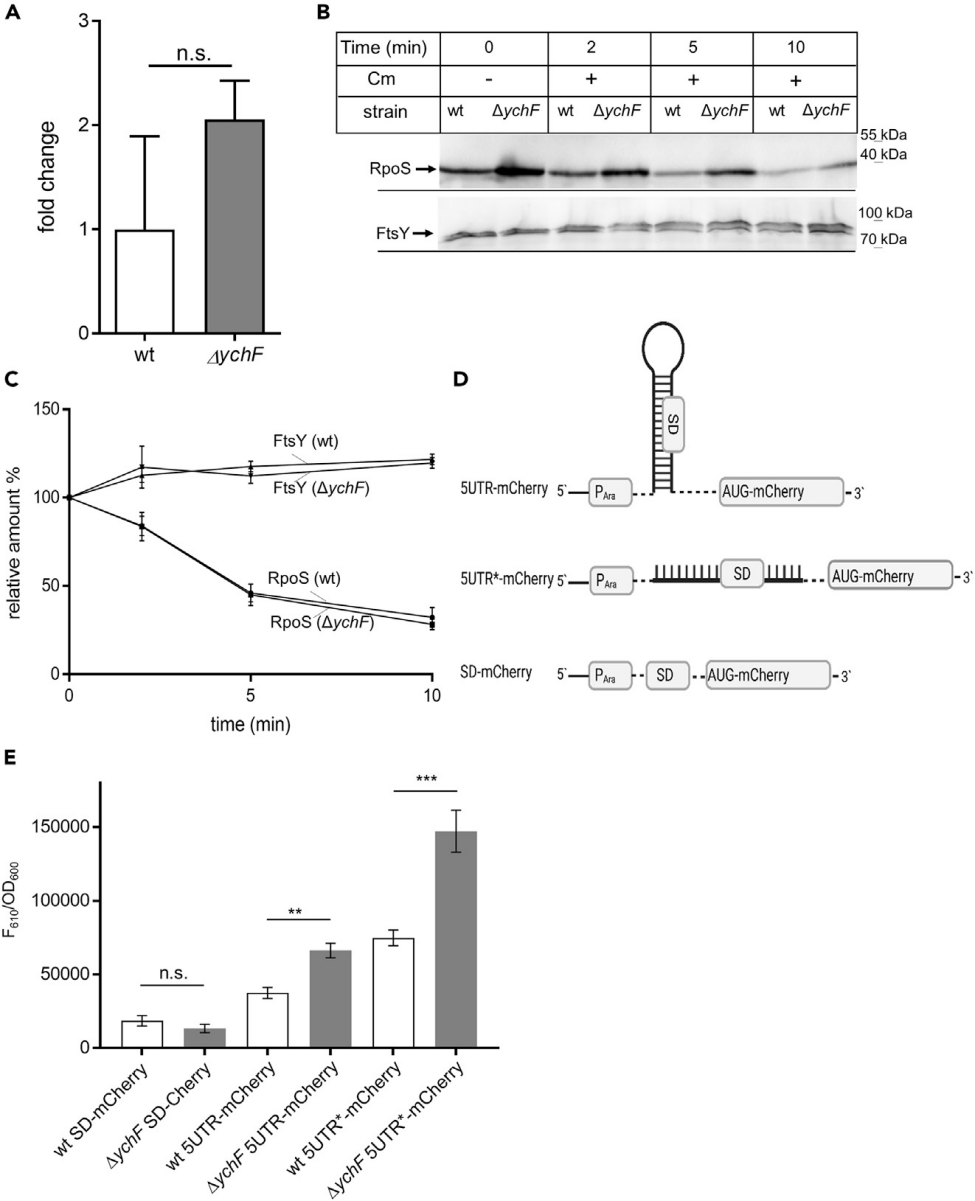
Post-transcriptional regulation of RpoS levels by YchF (A) The *rpoS* mRNA levels were determined and analyzed as described in Figure 1E from cells grown on LB medium to OD_600_ 0.8 (*n* = 3). (B) Wt and the Δ*ychF* strain were grown on LB medium up to OD_600_ 0.8 and protein synthesis was stopped by the addition of chloramphenicol (35 μg/mL). One sample was directly precipitated by TCA, while the others were only TCA precipitated after the indicated time points. Samples were then separated by SDS-PAGE and RpoS was visualized after western blotting. FtsY served as a control. (C) Quantification of the data shown in (B). Signal intensities of several independent experiments (RpoS *n* = 9; FtsY *n* = 3) were quantified by *ImageJ*. The values at time = 0 were set to 100%. (D) Cartoon depicting the translational mCherry fusion constructs for monitoring the influence of YchF on RpoS production. See text for details. (E) mCherry fluorescence of the constructs shown in (D) was monitored at an emission wavelength of 610 nm in wt and ΔychF cells grown in LB medium up to OD_600_ = 1.0 using a Tecan Spark plate reader. The values were corrected for the OD_600_ values and the F_610_/OD_600_ values are displayed. Values represent the mean values of *n* ≥ 3and the standard deviation is indicated. To determine the significance of the results, the *p* value was calculated using the “unpaired, two-tailed t test” of the program GraphPad prism. The *p* values are depicted as asterisks (∗) above the graphs as following: n.s. = *p* > 0.05; ∗∗ = *p* ≤ 0.01; ∗∗∗ = *p* ≤ 0.001.

The increase of RpoS in the absence of YchF could be the result of increased transcription, increased translation, or reduced degradation.^9^ Determining the *rpoS* mRNA levels by qRT-PCR did not show a significant increase in the Δ*ychF* strain compared to the wild-type strain (Figure 2A), which indicates that the absence of YchF does not drastically influence *rpoS* transcription.

In exponentially growing *E. coli* cells, RpoS is rapidly degraded by the ClpXP protease with the help of RssB as a recognition factor^58^^,^^59^ and YchF could stimulate RpoS degradation, resulting in elevated RpoS levels when YchF is absent. This was analyzed in whole cells after blocking protein synthesis by the addition of chloramphenicol and monitoring the stability of RpoS over time by western blotting (Figure 2B). Although the initial RpoS levels in the Δ*ychF* strain were higher than in the wild type, the overall RpoS degradation rate was similar in wild-type and Δ*ychF* cells (Figure 2B). This was also confirmed by quantitative analyses setting the RpoS level at t = 0% to 100% (Figure 2C). As further control, we monitored the degradation of FtsY over time. FtsY acts as a receptor for signal recognition particle-dependent protein transport,^60^ and its levels remain largely constant over time in both wild-type and Δ*ychF* cells (Figures 2B and 2C), although its activity is inhibited by (p)ppGpp accumulation.^21^ These data demonstrate that the increased levels of RpoS in the absence of YchF are not the result of increased transcription or reduced degradation.

YchF is a ribosome-binding protein that inhibits the translation of some mRNAs,^40^^,^^41^^,^^43^ which could also be the case for *rpoS*. For monitoring *rpoS* translation, an arabinose-inducible, plasmid-encoded translational fusion was constructed, in which the 5′ UTR of *rpoS*, consisting of 606 nucleotides and containing the native Shine-Dalgarno sequence, was fused to mCherry (5UTR-mCherry) (Figure 2D). In the native 5′ UTR of *rpoS*, the Shine-Dalgarno sequence is shielded within a stem-loop sequence, which needs to be unfolded by the RNA chaperone Hfq and the small RNA DsrA to boost RpoS synthesis.^61^ Increased *rpoS* translation is also observed when the accessibility of the Shine-Dalgarno sequence is improved by mutating the stem loop.^45^ These mutations were introduced into 5UTR-mCherry reporter resulting in the 5UTR∗-mCherry construct. Finally, in the SD-mCherry construct, the native *rpoS* 5′ UTR of *rpoS* was absent and mCherry was translated from the plasmid-derived Shine-Dalgarno sequence (Figure 2D). This latter construct served as a general control for transcription and translation of the mCherry-coding sequence. Fluorescence measurements of the SD-mCherry containing *E. coli* cells revealed a small reduction of fluorescence in Δ*ychF* cells compared to wild-type cells (Figure 2E). This is in line with the overall reduced protein synthesis in Δ*ychF* cells, which has also been observed in metabolic labeling experiments.^3^ In contrast, when the mCherry-coding sequence was preceded by the 5′ UTR of *rpoS* (5UTR-mCherry), the Δ*ychF* cells showed an approx. 40% increase in fluorescence compared to wild-type cells (Figure 2E). The highest fluorescence was observed for the 5UTR∗-mCherry construct and again the fluorescence in the Δ*ychF* cells was approx. 50% higher than in wild-type cells. In summary, these data indicate that the absence of YchF increases the translation of the *rpoS* mRNA, which is in line with previous studies demonstrating that YchF suppresses the translation of stress-responsive mRNAs.^30^^,^^41^ The data furthermore indicate that YchF does not regulate *rpoS* translation by influencing the accessibility of the Shine-Dalgarno sequence.

### The Δ*ychF* strain shows increased polyP levels

The increased RpoS levels and the induction of the general stress response could potentially explain the stress resistance of the *E. coli* Δ*ychF* strain. However, sigma factor-controlled gene expression is specifically executed in bacteria^12^ and does not explain the stress resistance of eukaryotic Δ*ola1* cells.

**Figure 3:**
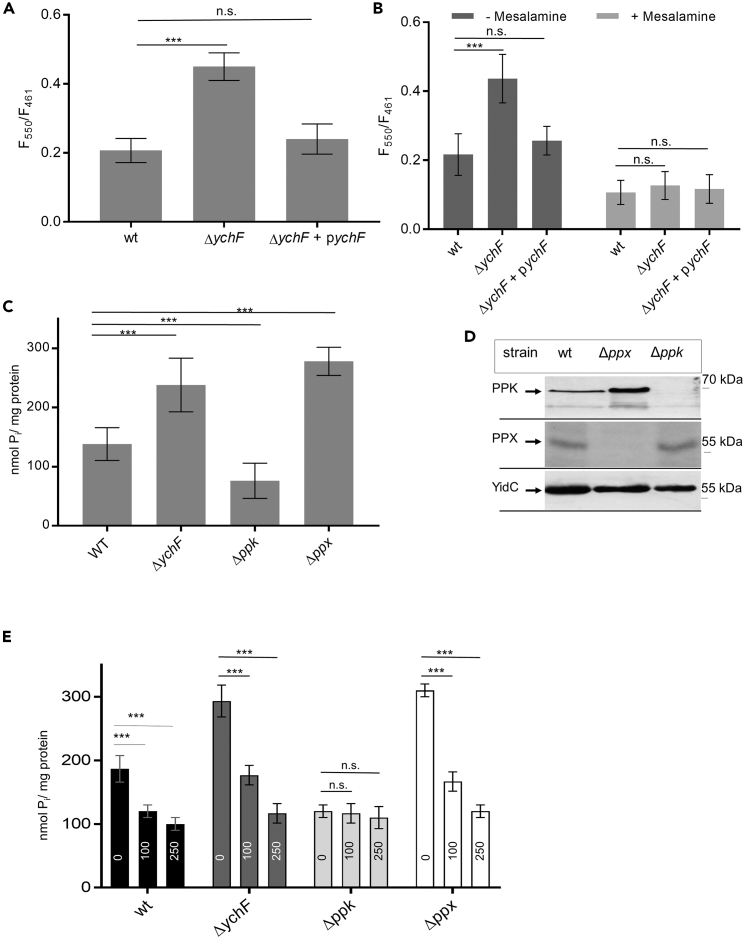
The absence of YchF increases the polyP levels in *E. coli* (A) Polyphosphate was determined in whole cells using DAPI fluorescence. Cells were grown overnight in LB medium, transferred to fresh LB medium, and grown up to an optical density of 0.6–0.7. Cells were then washed and transferred to minimal MOPS medium for 2 h. 2 × 10^8^ cells were then incubated with 10 μM DAPI for 5 min at 37°C. For polyP-DAPI, the fluorescence was determined after excitation at 414 nm and emission at 550 nm with the Tecan Spark plate reader. As internal control, the DNA-DAPI fluorescence was also determined after excitation at 358 nm and emission at 461 nm. Shown are the mean values of the F_550_/F_461_ values and standard deviations of *n* = 4 independent experiments. (B) As in (A), but polyP was also determined in cells that were incubated for 2 h in minimal MOPS medium in the presence of 200 μM mesalamine for inhibiting polyphosphate kinase (PPK). (C) Polyphosphate determination in cell extracts via *E. coli* exopolyphosphatase (PPX)-based polyP degradation and phosphate determination by malachite green. Cells were grown as above and shifted to minimal MOPS medium for 2 h. PolyP was then extracted from *E. coli* cells by phenol-chloroform treatment and precipitated by ethanol-sodium acetate treatment. After dissolving polyP, purified PPX (3 μM) was added and the released phosphate was determined spectrophotometrically by malachite green and the phosphate concentration was determined using a phosphate standard curve (0–40 μM). The phosphate concentrations were normalized to the protein content of the cell extract. Indicated are mean values and the standard deviations of *n* ≥ 5 independent experiments. (D) Immune detection of PPK and PPX in the corresponding single deletion strains. *E. coli* cells grown as above in MOPS minimal medium were TCA precipitated and after separation by SDS-PAGE and western blotting analyzed by the indicated antibodies. Shown is a representative blot of *n* = 3 repeats. (E) PPX-based polyP determination of mesalamine-treated *E. coli* cells. Cells were incubated with increasing mesalamine concentrations (0, 100, and 250 μM, as indicated within the columns) and analyzed as described in (C). The significances of the quantitative results were determined via the “unpaired, two-tailed t test” of the program GraphPad prism. The *p* values are depicted as asterisks (∗) above the graphs as following: n.s. = *p* > 0.05; ∗∗∗ = *p* ≤ 0.001.

One universally conserved strategy of stress protection in both bacteria and eukaryotes is the production of polyP, which acts as a chemical chaperone and prevents stress-induced protein aggregation.^62^^,^^63^ The recently observed increase of the vacuolar transporter chaperone complex (VTC) in yeast Δ*ola1* cells,^31^ which is required for vacuolar polyP accumulation,^64^ provides a direct link between YchF/Ola1 and polyP production. Furthermore, polyP has been shown to induce RpoS production.^65^^,^^66^ We therefore determined the polyP levels *in vivo* by DAPI (4′,6-diamino-2-phenylindole) fluorescence. DAPI binding to polyP shifts its emission wavelength from 475 to 550 nm after excitation at 414 nm.^67^ Fluorescence emission of free DAPI or DNA-bound DAPI is minimal at these wavelengths, enabling the specific detection of polyP *in vivo*. Fluorescence measurements revealed that the Δ*ychF* strain showed approx. 2-fold increased DAPI fluorescence when compared to the wild type (Figure 3A), suggesting that the absence of YchF leads to increased polyP production. This was verified in a Δ*ychF* strain expressing a plasmid-borne *ychF* copy, which showed a DAPI fluorescence that was comparable to the one in wild-type strains.

The majority of polyP in *E. coli* is synthesized by polyphosphate kinase (PPK), although it has been suggested that other kinases might also contribute to the cellular polyP levels.^14^^,^^68^^,^^69^ The salicylic acid derivative mesalamine inhibits PPK-catalyzed polyP production,^70^ and the polyP levels were therefore tested in mesalamine-treated cells. Mesalamine treatment reduced DAPI fluorescence in all three strains to a comparable level, suggesting that the increased polyP levels in the Δ*ychF* strain are likely the result of increased PPK activity (Figure 3B).

Although DAPI staining is a very fast and simple method for determining the polyP levels in whole cells, red-shifted DAPI emission has also been observed with RNA, calcium phosphate, or inositol phosphates at high DAPI concentrations.^71^^,^^72^ We therefore employed an additional assay for polyP determination. PolyP was extracted from whole cells by phenol-chloroform treatment and subsequently degraded by purified *E. coli* exopolyphosphatase (PPX), which is responsible for degradation of long-chain polyP in *E. coli*.^73^ The PPX-catalyzed release of phosphate was then determined spectrophotometrically by malachite green. This enzymatic assay revealed approx. twice as much polyP in the Δ*ychF* compared to the wild-type strain (Figure 3C). The polyP levels were also determined in Δ*ppk* and Δ*ppx E. coli* strains. The Δ*ppk* strain showed reduced polyP levels, although some polyP was still detectable, either because small amounts of polyP are produced by other kinases^62^ or due to some phosphate contamination in the assay. On the other hand, in the Δ*ppx* strain, the polyP levels were approx. twice as high as in the wild type, which is in line with the inability of the Δ*ppx* strain to efficiently degrade polyP (Figure 3C). The polyP levels were determined in *E. coli* cells after shifting them to minimal MOPS medium,^74^ and we verified the absence of the PPX and PPK in the corresponding strains under those conditions by immune detection using peptide antibodies (Figure 3D). Interestingly, the Δ*ppx* strain showed increased levels of PPK (Figure 3D). *Ppk* and *ppx* are organized in one operon,^73^ and it has to be further analyzed why the absence of *ppx* increases the steady-state levels of PPK.

The PPX-based polyP assay was also used on cells that were treated with mesalamine before polyP extraction. Reduced polyP levels were observed in wild-type, Δ*ppx*, and Δ*ychF* strains when treated with increased mesalamine concentrations (0–250 μM, Figure 3E). This was not observed in the Δ*ppk* strain, supporting a PPK-independent polyP production. In conclusion, the absence of YchF increases the concentration of the chemical chaperone polyP in *E. coli*.

### The lack of YchF does not influence the expression of the *ppk-ppx* operon

**Figure 4:**
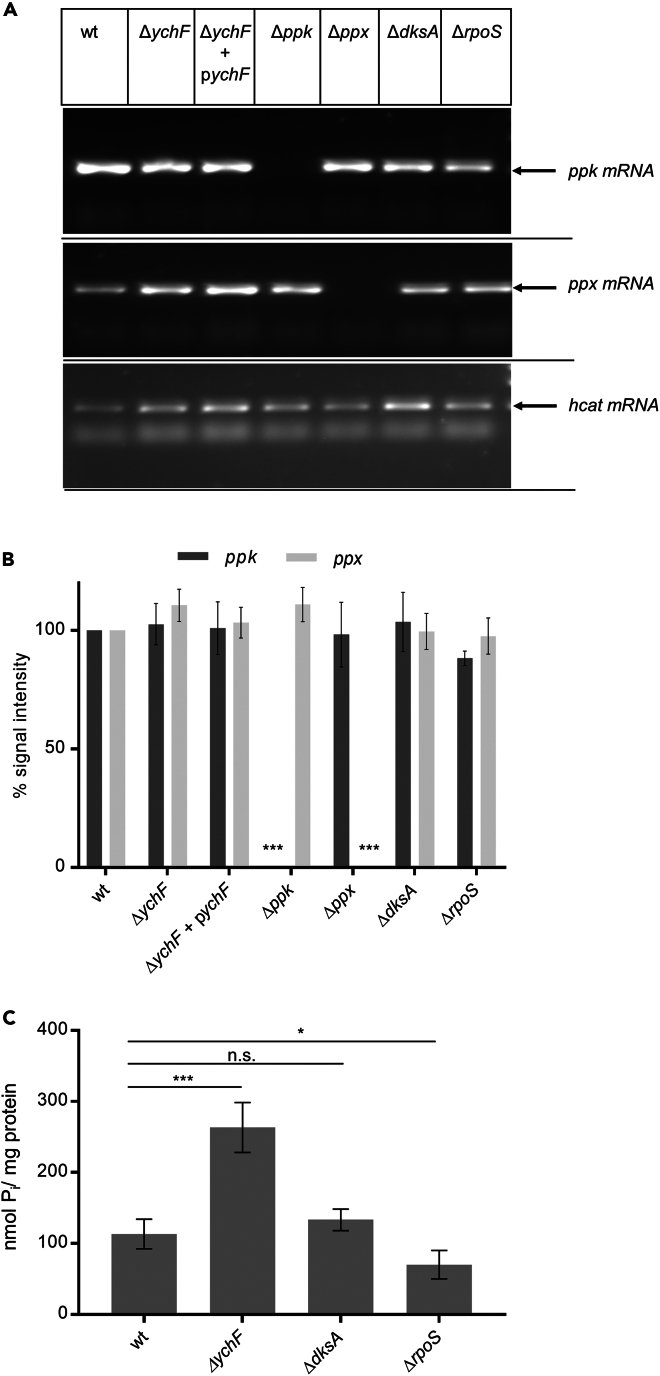
YchF does not influence the transcription of the *ppk-ppx* operon (A) Total mRNA was isolated from the indicated strains and treated as described in Figure 1, followed by RT-PCR using specific *ppk*, *ppx*, and *hcat* primer, respectively. (B) Quantification of the data shown in (A). Shown are the mean values and standard deviations of *n* ≥ 4 independent experiments. For the sake of clarity, only significant *p* values are displayed. (C) PolyP was determined by the PPX-based assay as in Figure 3C and shown are the mean values and standard deviations of *n* ≥ 4 independent experiments. The significance of the results was determined via the “unpaired, two-tailed t test” of the program GraphPad prism. The *p* values are depicted as asterisks (∗) above the graphs as following: n.s. = *p* > 0.05; ∗ = *p* ≤ 0.05; ∗∗ = *p* ≤ 0.01; ∗∗∗ = *p* ≤ 0.001.

PolyP production in *E. coli* is regulated by RpoS and the transcription factor DksA,^14^ but also by the sigma factors RpoE and RpoN.^75^ It therefore seemed possible that the absence of YchF and the simultaneously increased levels of RpoS stimulate transcription of the *ppk-ppx* operon and as a consequence polyP production. The *ppk* and *ppx* mRNA levels were determined by reverse-transcription PCR (RT-PCR) using the *hcaT* mRNA as a control. The absence of *ychF* or the presence of a plasmid-borne copy of *ychF* had no detectable influence on the *ppx* or *ppk* mRNA levels (Figure 4A), which indicated that neither the absence of YchF nor the increase of RpoS had a major influence on the transcription of the *ppk-ppx* operon. The specificity of the used RT-PCR primers was confirmed by using the mRNAs of the *ppk* and *ppx* strains for RT-PCR.

The *ppk-ppx* mRNA levels were also determined in Δ*rpoS* and Δ*dksA* strains; however, the lack of RpoS or DksA did not result in significant changes in the *ppk* or *ppx* mRNA levels (Figure 4A). The quantification of the RT-PCR data (*n* ≥ 4) by *ImageJ* and using *hcaT*-normalized wild-type mRNA levels as a control (=100%) showed only small variations of the mRNA levels in the different strains but confirmed the absence of *ppk* or *ppx* in the corresponding Δ*ppk* and Δ*ppx* strains (Figure 4B). This is in agreement with previous reports showing that although DksA and RpoS influence the polyP levels in *E. coli*, they do not influence transcription of the *ppk-ppx* operon.^14^^,^^75^^,^^76^

Next, the polyP levels were determined in the Δ*rpoS* and Δ*dksA* strains, which revealed comparable polyP levels in in the wild-type and the Δ*dksA* strains and slightly reduced polyP levels when *rpoS* was missing (Figure 4C). This is in conflict with previous data, which showed a clear effect of RpoS and DksA on polyP production.^14^^,^^76^ This likely reflects differences in the polyP metabolism between different *E. coli* strains. While in our study *E. coli* BW25113 was used as parental strain, previous studies analyzed the polyP levels in *E. coli* MG1665.^14^^,^^75^^,^^76^

In conclusion, although the polyP levels are increased in the absence of YchF, this is not the result of changes in the transcription of *ppk* or *ppx*.

### YchF regulates the steady-state levels of PPK

**Figure 5:**
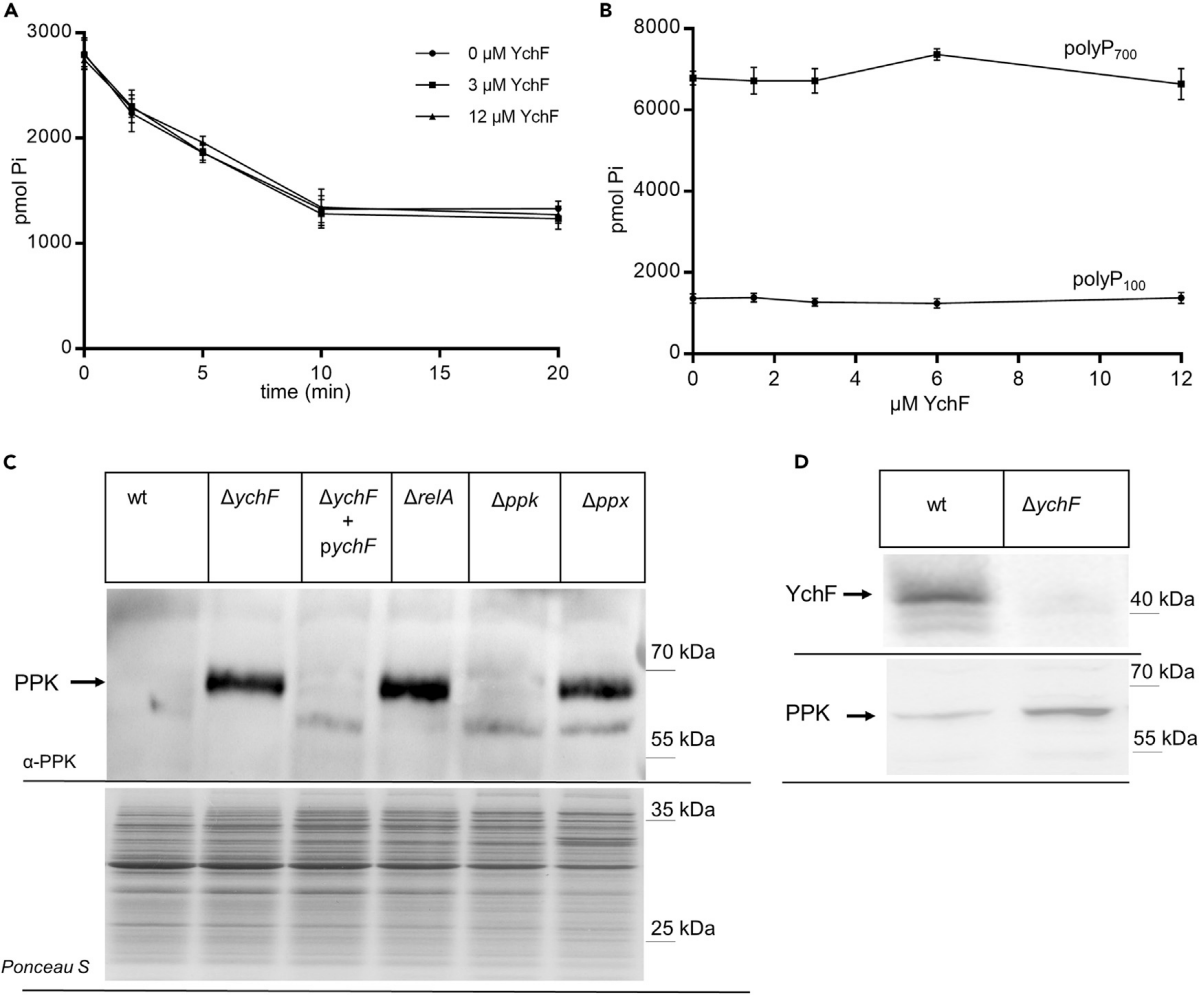
YchF increases the steady-state levels of PPK (A) *In vitro* assay of PPX-based polyP_100_ degradation. Purified PPX (3 μM) was incubated with 1 mM polyP_100_ in the absence or presence of purified YchF (3 μM or 12 mM final concentration). At the indicated time points, samples were taken and the released phosphate was determined spectrophotometrically by malachite green. Shown are the mean values of three independent experiments and the standard deviation is indicated by error bars. (B) The *in vitro* assay was performed as in (A), but as endpoint assay (20 min incubation, *n* = 3) in the presence of different YchF concentrations and with polyP_100_ and polyP_700_ (1 mM final concentration) as substrates. (C) Immune detection of PPK in different strains. Cells were grown in LB medium and 2 × 10^8^ cells were TCA precipitated, and separated by SDS-PAGE. Note, that the PPK levels in wt cells grown on LB medium are below the detection level. After western blotting the upper part of the membrane was analyzed with PPK antibodies and the lower part of the membrane was stained with *Ponceau S*. Shown is a representative blot of *n* = 3 repeats. (D) Wt and Δ*ychF* cells were grown on LB medium to an optical density of 0.8 and then shifted to minimal MOPS medium for 2 h before TCA precipitation, SDS-PAGE, and immune detection using α-YchF and α-PPK antibodies. Shown is a representative blot of *n* = 3 repeats.

The aforementioned data indicate that YchF controls the polyP levels by a post-transcriptional mechanism. YchF could potentially stimulate the activity of PPX, which in turn would lead to reduced PPX activity and higher polyP levels when YchF is absent. This was determined by measuring time-dependent polyP degradation by PPX in the absence or presence of purified YchF. PPX-dependent degradation of polyP with a chain length of 100 (PolyP_100_) was not influenced by the addition of equimolar concentrations of YchF (3 μM). Even at a 4-fold excess, YchF did not inhibit polyP degradation (Figure 5A). Since *E. coli* PPX preferentially hydrolyses long-chain polyP,^73^ we also tested the effect of different YchF concentrations on PolyP_100_ and PolyP_700_ degradation in an endpoint assay. These assays validated that YchF does not influence polyP degradation by PPX *in vitro* (Figure 5B). Thus, it appears unlikely that YchF controls the polyP levels by stimulating the activity of PPX.

Alternatively, the absence of YchF could increase the activity or steady-state levels of PPK and this was analyzed by immune detection of PPK in whole cells using peptide antibodies. The detection of PPK in wild-type cells grown on LB medium was generally difficult, which is likely explained by the low copy number of PPK in wild-type *E. coli* cells (approx. 250 copies/cell^77^). In agreement, the antibody failed to detect PPK in LB-grown wild-type cells (Figure 5C). However, in the Δ*ychF* strain, the antibody recognized a strong band at approx. 70 kDa, which is in line with the experimentally verified molecular mass of PPK^68^ (Figure 5C). In the Δ*ychF* strain expressing a plasmid-borne *ychF* copy, PPK was not detectable, further supporting that the absence of YchF increases the steady-state levels of PPK.

The alarmones (p)ppGpp were suggested to regulate the polyP levels in *E. coli* by inhibiting PPX,^78^ and although this was questioned in a recent study,^14^ polyP has been shown to accumulate in a Δ*relA* mutant,^14^^,^^69^ which is impaired in (p)ppGpp synthesis.^79^ However, whether the Δ*relA* strain has increased levels of PPK, as observed in the Δ*ychF* strain, was not tested so far. Only for a Δ*relA*-Δ*spoT* double mutant, a lower *ppk* expression was observed based on promoter fusion experiments.^14^ Our data now show that indeed the PPK steady-state levels increase when RelA is absent (Figure 5C), which potentially explains the increased polyP levels in this strain. The PPK levels also increase when *ppx* is deleted, which is also seen for MOPS-shifted cells (Figure 3D). The reason for this is currently unknown but apparently not directly linked to differences in mRNA stability (Figures 4A and 4B).

In order to clearly link the increased polyP production in the Δ*ychF* strain to the increased PPK levels, the PPK levels were also determined after shifting cells to MOPS minimal medium. As seen for LB-grown cells, the PPK levels increased in the absence of YchF (Figure 5D). This demonstrates that the absence of YchF increases the PPK levels in *E. coli* which in turn leads to increased polyP production.

The increased PPK levels in the absence of YchF are not linked to differences in the *ppk* mRNA levels, which is also observed for RpoS and its mRNA. This provides further support for a post-transcriptional function of YchF, which is most likely linked to its ability to interact with ribosomes and regulate translation.^3^^,^^24^^,^^40^^,^^41^

### The *ychF-ppx* double mutant is synthetically lethal

**Figure 6:**
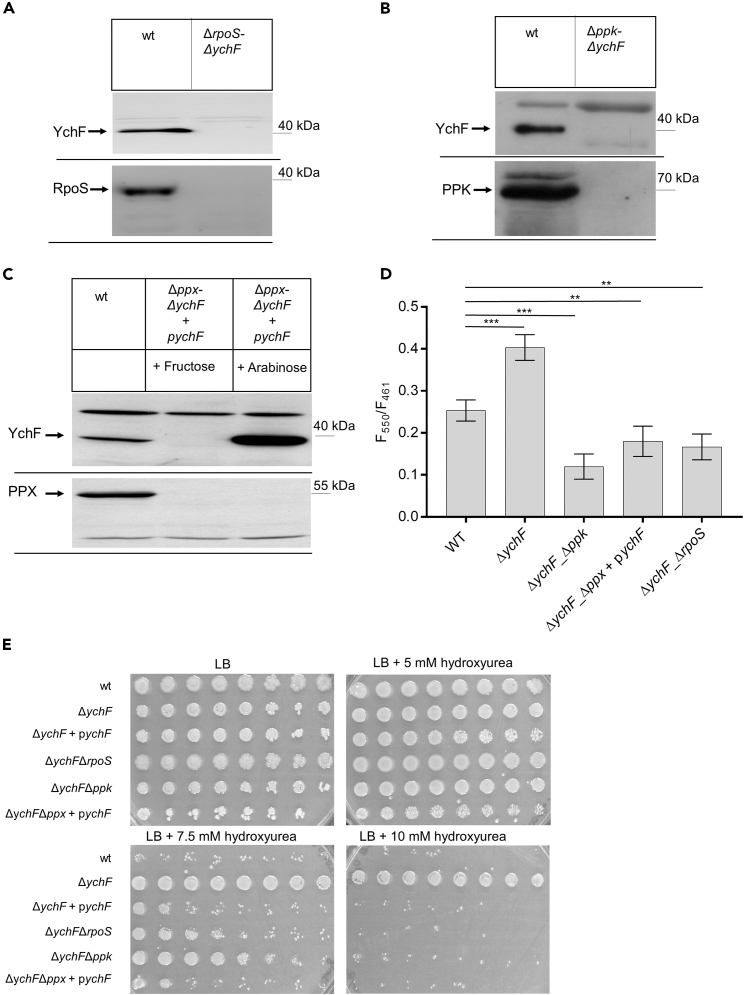
Synthetic lethality of a *ychF-ppx* double knockout strain (A) A *ychF-rpoS* double knockout strain was generated via λ-red recombination, grown on LB medium, and analyzed with α-YchF and α-RpoS antibodies. (B) The *ychF-ppk* double knockout strain was also generated via λ-red recombination and whole cells were analyzed by immune detection using α-YchF and α-PPK antibodies after cell growth for 2 h on MOPS minimal medium. (C) Deleting *ppx* in a Δ*ychF* background was only possible in a Δ*ychF* strain containing a *ychF* copy on the pBAD24 plasmid. However, Δ*ychF*Δ*ppx* + p*ychF* cells were able to grow on LB medium even in the absence of the inducer arabinose, because the *ara* promoter showed some leakiness. (D) Polyphosphate levels in the indicated strains were determined via DAPI staining of cells grown first on LB medium, before shifting them to MOPS minimal medium, as described in Figure 3 (*n* = 4). The significance of the results was determined via the “unpaired, two-tailed t test” of the program GraphPad prism. The *p* values are depicted as asterisks (∗) above the graphs as following: n.s. = *p* > 0.05; ∗ = *p* ≤ 0.05; ∗∗ = *p* ≤ 0.01; ∗∗∗ = *p* ≤ 0.001. (E) The indicated strains were grown on LB medium or on LB medium containing 0.01% arabinose in case of Δ*ychF*Δ*ppx* + p*ychF* to OD_600_ of ∼1.0, washed once with PBS before serial dilution in PBS. Of each dilution, starting with 10^7^ cells, 10 μL cell suspensions were spotted onto LB plates and LB plates containing, 5, 7.5, or 10 mM hydroxyurea. Cell growth was monitored after overnight incubation at 37°C. See also Figure S2.

The link between RpoS, polyP production, and YchF was further confirmed by generating *ychF-rpoS*, *ychF-ppk*, and *ychF-ppx* double knockout strains using λ-red recombination.^80^ While *ychF-rpoS* and *ychF-ppk* double mutants were readily observed and validated by PCR and western blotting using the Δ*ychF* strain as a host (Figures 6A and 6B), several attempts to generate a *ychF-ppx* double mutant failed. As control, a Δ*ppx* single knockout strain was generated using *E. coli* BW25113 as host strain, which is the parental strain of the Δ*ychF* strain. Thus, it appeared likely that the simultaneous absence of YchF and PPX is causing synthetic lethality and this was verified by deleting *ppx* in the Δ*ychF* strain that contained a plasmid-borne copy of *ychF* under control of the arabinose promoter. In this strain, *ppx* could be deleted and further growth experiments demonstrated that optimal growth of the Δ*ychF*Δ*ppx* strain was only possible in the presence of arabinose for inducing the plasmid-borne *ychF* copy. However, weak growth of this strain on LB medium was even possible in the presence of fructose, likely because the *ara* promoter is not completely tight (Figure 6C). In conclusion, these data indicate that the increased polyP production in the absence of YchF is only tolerated in the presence of the polyP-degrading enzyme PPX. Due to this PPX-based degradation, the absence of YchF boosts polyP production likely to a much higher level than our assays suggest.

The polyP levels in these double knockouts were determined in whole cells using DAPI staining. In the *ychF-ppk* double knockout, the polyP levels were much lower than in the Δ*ychF* strain, further validating that the higher polyP levels in the Δ*ychF* strain are the result of increased PPK production (Figure 6D). In the Δ*ychF*Δ*ppx* strain containing the p*ychF* plasmid, the polyP levels were also lower than in wild-type or Δ*ychF* cells, which confirms that increased levels of YchF prevent polyP accumulation. Finally, the polyP levels in the Δ*ychF*Δ*rpoS* strain were lower than in Δ*ychF* cells. This is in line with the reported influence of RpoS on polyP production, which includes both transcriptional^81^ and post-transcriptional^14^^,^^75^^,^^76^ effects and which is also seen in the reduced polyP levels of the Δ*rpoS* single knockout (Figure 4C).

The absence of YchF has been shown to increase stress resistance, which is exemplified by the resistance of the Δ*ychF* strain against the replication inhibitor hydroxyurea (HU)^41^^,^^82^ (Figure 6E). While wild-type cells showed strongly reduced growth in the presence of 10 mM HU, the growth of the Δ*ychF* strain was unaffected, unless it contained a plasmid-borne *ychF* copy. The Δ*ychF*Δ*rpoS* strain was as sensitive as the wild type and the same was observed for the Δ*ychF*Δ*ppx* strain containing the p*ychF* plasmid. The sensitivity of the Δ*ychF*Δ*rpoS* strain was expected due to the lower levels of polyP. In addition, HU not only inhibits ribonucleotide reductase but has also been suggested to increase the formation of reactive oxygen species,^83^ which require the RpoS-induced production of the catalase KatE or the alkyl peroxidase reductase AhpF.^81^ Finally, also the Δ*ychF*Δ*ppk* strain showed the same sensitivity as the wild-type strain, suggesting that indeed the HU resistance of the Δ*ychF* strain is dependent on increased polyP levels. As controls, we also monitored the HU sensitivity of the single knockout strains. The Δ*ppx* strain showed that the Δ*ychF* strain increased resistance toward 7.5 mM HU but did not grow in the presence of 10 mM HU. The other strains were as sensitive as the wild-type strain (Figure S2). Thus, the HU resistance of *E. coli* is apparently the result of both increased polyP levels and the RpoS-induced stress response.

## Discussion

Despite the high sequence conservation and the ubiquitous distribution in all domains of life, a unifying concept on the molecular functions of YchF/Ola1 is missing.^3^^,^^24^^,^^29^ Still, the available data indicate that one potentially conserved function of YchF/Ola1 is its ability to interact with ribosomes and control the translation of stress response proteins.^30^^,^^31^^,^^40^^,^^41^^,^^42^^,^^43^ YchF/Ola1 is furthermore considered to be a constituent of the minimal translation machinery,^84^ although the deletion of its gene is tolerated in bacterial, yeast, and mammalian cells.^27^^,^^31^^,^^85^ The deletion of *ychF* in *E. coli*^27^^,^^39^ or the depletion of Ola1 in human cell lines^28^^,^^33^ rather leads to an unusual gain-of-function phenotype, because these cells show increased resistance toward stress conditions.^28^^,^^86^ In *E. coli*, the absence of YchF increases catalase activity,^27^ and it was initially suggested that YchF might act as a catalase inhibitor, a hypothesis that is further supported by site-directed *in vivo* cross-linking experiments, which found catalases as YchF-interacting proteins.^27^^,^^39^ However, *in vitro* data did not show any inhibitory effect of YchF on catalase KatG,^41^ which is the main catalase in *E. coli*, leaving it largely open, how YchF regulates the oxidative stress response.

Our data now demonstrate that the absence of YchF increases the levels of the transcriptional regulator RpoS, which in turn induces the expression of KatE, which acts as catalase during the stationary phase.^51^ This explains the increased catalase activity in Δ*ychF* cell extracts and the oxidative stress tolerance. The RpoS regulon in *E. coli* comprises more than 500 genes, many of which are involved in the adaptation to stress conditions and starvation.^11^ During the exponential phase, RpoS in *E. coli* is kept at a low concentration by a complex network of proteases and transcriptional and translational regulators.^45^^,^^46^^,^^58^^,^^61^^,^^87^ YchF likely acts as an additional translational regulator that prevents the accumulation of RpoS during the exponential phase. This is in line with data showing that YchF prevents the translation of stress-responsive mRNAs under non-stress conditions and that the absence of YchF in *E. coli* or Ola1 in eukaryotes stimulates alternative translation initiation mechanisms that increase the production of stress response proteins.^30^^,^^41^ The coding sequence of *rpoS* is preceded by a 606-nucleotide-long 5′ UTR containing several stem loops which also shield the ribosome-binding site.^88^ However, the mCherry reporter fusion experiments revealed that the absence of YchF increases translation even when the stem loop shielding the ribosome-binding site is mutated. This indicates that YchF does not regulate translation by preventing the unfolding of the stem loop, which could be envisioned based on the observation that YchF binds nucleic acids.^89^ The stability and translation of the *rpoS* mRNA are determined by multiple factors in *E. coli*. The small RNAs DsrA and RprA stabilize *rpoS* and stimulate translation and a similar effect is executed by Hfq.^9^^,^^18^ On the contrary, H-NS and LrhA reduce *rpoS* translation.^90^^,^^91^ It is currently unknown whether YchF influences the abundance or activity of these factors, but the reduction of the YchF levels when cells enter stationary phase^41^ stimulates RpoS production and the induction of the RpoS regulon. The LysR-type transcriptional regulator OxyR downregulates *ychF* expression and induces the oxidative stress response.^27^^,^^92^^,^^93^ Intriguingly, *oxyR* expression is negatively regulated by RpoS^94^ and this could provide a regulatory circuit for fine-tuning the oxidative and starvation stress responses.

In addition to the increased RpoS level, our data also showed increased levels of polyP in the Δ*ychF* strain. RpoS and polyP metabolism are intricately linked *in E. coli*: transcription of the *ppk-ppx* operon is regulated by RpoS^81^ and increased polyP levels have been shown to stimulate *rpoS* transcription.^66^ The qRT-PCR data, however, do not show significant differences in the *rpoS* mRNA levels between the wild-type and Δ*ychF* strain. Thus, the increase of the polyP levels appears to be insufficient for stimulating *rpoS* transcription. Nevertheless, the induction of the RpoS regulon nicely explains the increased stress resistance of the *E. coli* Δ*ychF* strain. However, as sigma factor-based gene regulation is typical for bacteria,^12^ it does not explain the increased stress resistance of eukaryotic Δ*ola1* cells.

The evolutionary ancient biopolymer polyP could provide the link between the observed stress resistance of eukaryotic Δ*ola1* and prokaryotic Δ*ychF* cells. PolyP is a universally conserved and multi-functional chemical polymer^70^^,^^95^^,^^96^ that acts as a chaperone, sequesters heavy metals, serves as phosphate storage, and influences DNA repair and inflammation.^97^^,^^98^ Furthermore, polyP increases the oxidative stress and temperature resistance in both prokaryotes and eukaryotes.^95^^,^^99^^,^^100^ Our data demonstrate that in the absence of YchF, the steady-state levels of the polyP-synthesizing enzyme PPK strongly increase, which explains the high polyP levels. We furthermore show that the polyP levels in the Δ*ychF* strain are sensitive to the PPK inhibitor mesalamine and that deleting both *ychF* and *ppk* diminishes stress resistance. We did not observe significant differences in the *ppk* mRNA levels, suggesting that the absence of YchF enhances the synthesis of PPK, similar to the stimulated synthesis of RpoS. The coding sequences of both *rpoS* and *ppk* are preceded by long 5′ UTRs, but we did not find any indication that YchF prevents the unfolding of the *rpoS* stem loop and thus blocks the accessibility of the ribosome-binding site. However, this could be of course different for the stem loop preceding the *ppk* coding sequence. Post-translational regulation of PPK in *E. coli* can also influence the polyP levels, but this does not substantially influence the PPK levels,^101^ which is different from what we observe here. Nevertheless, we cannot exclude that YchF also influences the PPK activity.

Intriguingly, the simultaneous deletion of *ychF* and the polyP-degrading enzyme PPX causes synthetic lethality, which suggests that high levels of polyP are toxic to *E. coli*, which has also been observed in humans.^102^ The simultaneous deletion of *ychF* and *ppx* likely induces Mg and phosphate starvation.^101^ Phosphate starvation in turn stimulates RpoS accumulation,^103^ which provides another link between YchF, RpoS, and polyP production. The necessity to keep the polyP levels within a certain physiological range potentially explains why the production of polyP is controlled by YchF as a negative regulator and why the Δ*ychF* strain is outcompeted by the wild-type under non-stress conditions.^41^

One hallmark of human *OLA1* is that it is upregulated in many cancer cells and that both metastasis and long-term survival are influenced by the Ola1 levels.^33^^,^^38^^,^^104^^,^^105^^,^^106^^,^^107^ The polyP levels in humans have been shown to stimulate multiple signaling pathways, including the mTOR pathway,^95^^,^^97^ and decreased levels of polyP have been linked to several pathological conditions, such as cancer.^98^ Thus, Ola1 and YchF could both act via regulating polyP homeostasis, which would reconcile many of the observed phenotypes in prokaryotes and eukaryotes and provide a first hint for a common mechanism of these conserved ATPases. Although, there is only limited knowledge about polyP synthesis and degradation in mammals,^108^ the recently observed upregulation of the polyP-producing VTC complex in yeast cells when Ola1 is deleted^31^ suggests that indeed the regulation of polyP production is a conserved function of YchF/Ola1.

### Limitations of the study

Our data demonstrate that *E. coli* YchF controls the production of RpoS and PPK by a transcription-independent mechanism. Although this is supported by data showing that YchF interacts with ribosomes and inhibits translation of stress-responsive mRNAs, details about the underlying mechanism are still missing. Thus, further studies need to explore how YchF/Ola1 selectively inhibits translation of some mRNAs and whether this is linked to ribosome heterogeneity in different species. In addition, with respect to the regulation of polyP metabolism in *E. coli*, it is important to emphasize that there are strain-dependent differences.

## STAR★Methods

### Key resources table

**Table:** 

REAGENT or RESOURCE	SOURCE	IDENTIFIER
**Antibodies**		

α-YchF	Wenk et al., 2012^27^	N.A.
α-RpoS, monoclonal antibody, clone 1RS1	Biolegend	Cat#663703
α-PPK, peptide antibodies from rabbits	This study	N.A.
α-YidC, rabbits	Koch et al., 2002^109^	N.A.
α-PPX peptide antibodies from rabbits	This study	N.A.
α-FtsY, rabbits	Koch et al., 1999^110^	N.A.
α-His-HRP	Thermo Scientific	Cat#15165
α-rabbit IgG	Caltag Medsystems	Cat#DL87009A

**Bacterial and virus strains**		

*E. coli* DH5α	Hanahan 1983^111^	N.A.
*E. coli* BL21	Merck, Darmstadt, Germany	Cat#69449
*E. coli* C43(DE3)	Miroux & Walker, 1996^112^	N.A.
*E. coli* (Δ*ychF* Km^S^)	Wenk et al., 2012^27^	JW1194
*E. coli* (Δ*ppk*)	KEIO Collection	JW2486
*E. coli* (Δ*ppx*)	KEIO Collection	JW2487
*E. coli* (Δ*rpoS*)	KEIO Collection	JW5437
*E. coli* (Δ*dksA*)	KEIO Collection	JW0141
*E. coli* (Δ*relA*)	KEIO Collection	JW2755
*E. coli* BW25113	Datsenko & Wanner, 2000^80^	N.A.

**Biological samples**		

YchF	This study, Wenk et al., 2012^27^, Landwehr et al., 2021^3^^,^^41^	N.A.
PPX	This study	N.A.
PPK	This study	N.A.

**Chemicals, peptides, and recombinant proteins**		

^35^S-Methionine labeling mix	Hartmann Analytics Braunschweig, Germany	Cat#ARS0110
YchF peptide VNEDGFENNPYLDQC	Genescript, Rijswijk, Netherlands	N.A.
PPK peptide IIISEEQGSNSHSRC	Genescript, Rijswijk, Netherlands	N.A.
PPX peptide VEHTQPEKGRKLVIC	Genescript, Rijswijk, Netherlands	N.A.
RNAsin Ribonuclease Inhibitor	Promega, Mannheim, Germany	Cat#N2515
RNA loading dye	NEB	Cat#B0363S
DEPC-treated H_2_O	Thermo Scientific	Cat#AM9915G
PolyP100	Kerafast	EUI005
PolyP700	Kerafast	EUI002
DAPI	Sigma Aldrich	D9542-5 MG
Mesalamine	Sigma Aldrich	PHR1060
Talon metal affinity resin	Takara/Clontech	Cat#635503

**Critical commercial assays**		

T4-Polynucleotide-Kinase	Thermo-Fisher	Cat#EK0031
Gibson HiFi Assembly Master Mix	NEB	Cat#E2611L
Q5 hot start high-fidelity 2X master mix	NEB	Cat#M0494S
KLD enzyme mix	NEB	Cat#M0554S
RevertAid reverse transcriptase	Thermo-Fisher	EP0441
Ribolock RNAse inhibitor	Thermo-Fisher	EO0382
Phosfinity ChainQuant Kit	Aminoverse B.V. Netherlands	–
Q5 Site directed mutagenesis	NEB	Cat#E0554S
Malachite Green Phosphate Assay Kit	Sigma-Aldrich	MAK307-1KT
QIAquick PCR purification Kit (50)	QIAGEN	Cat.No.28104
QIAprep Spin Miniprep Kit (250)	QIAGEN	LOT 172041779
DNeasy Blood& Tissue Kit (50)	QIAGEN	Cat. No.69504
RNeasy Mini Kit (50)	QIAGEN	Cat. No.74104

**Oligonucleotides**		

*see*STAR methods*section*		

**Recombinant DNA**		

*See*STAR methods*section*		

**Software and algorithms**		

*ImageJ/Fiji*	National Institute of Health	https://imagej.nih.gov/ij/index.html
*ImageQuant TL10.2*	Cytiva Europe GmbH	https://www.cytivalifesciences.com/en/us/shop/molecular-biology/nucleic-acid-electrophoresis--blotting--and-detection/molecular-imaging-for-nucleic-acids/imagequant-tl-10-2-analysis-software-p-28619
GraphPad Prism	GraphPad Prism Corp. USA	https://www.graphpad.com/scientific-software/prism/
Welch-test		https://matheguru.com/

### Resource availability

#### Lead contact

Further information and requests for resources and reagents should be directed to the lead contact, Hans-Georg Koch (Hans-Georg.Koch@biochemie.uni-freiburg.de).

#### Materials availability

All plasmids are available upon request, subject to a material transfer agreement (MTA), from Hans-Georg Koch (Hans-Georg.Koch@biochemie.uni-freiburg.de).

#### Data and code availability

• All data reported in this paper will be shared by the lead contact upon request.
• This paper does not report original code.
• Any additional information required to reanalyze the data reported in this paper is available from the lead contact upon request.

### Experimental model and subject details

All bacterial strains used in this study are derived from wild type *E. coli* K-12 strain.^80^^,^^111^^,^^112^^,^^113^ *Escherichia coli* BW25113 was used as wild type *E.coli* strain and *E. coli* strain JW1194 (Km^S^) as Δ*ychF* strain.^27^ For *T7*-dependent expression, the *E. coli* strain C43(DE3) (Novagen/Merck, Darmstadt, Germany) was used. The *E. coli* strains: *Δppk, Δppx, ΔrpoS, ΔdksA, and ΔrelA* were from the Keio collection and were obtained from Thermo Fisher Scientific (Darmstadt, Germany); the parental strain for all deletions was *E. coli* BW25113. *E. coli* cells were routinely grown on LB-medium at 37°C in either liquid media or on solid media (LB medium +1.5% agar-agar) unless otherwise stated. All plasmids were constructed by using the HiFi DNA assembly Master Mix Gibson assembly protocol using 100 ng vector and a 1:3 vector/insert ratio. For mutagenesis, the NEB Site directed mutagenesis kit using the manufacture’s protocol was used. The 5′-UTRmCherry plasmid was constructed by BioCat GmbH (Heidelberg, Germany). The sequence of all plasmids was confirmed by sequencing.

### Method details

#### Construction of chromosomal knock-out strains

The double-knockout strain *ΔychFΔppk* was constructed by the λ-Red recombination method,^80^ using the *ΔychF* strain containing the plasmid pKD46^80^ as parent strain and the oligonucleotides ppk-left 5′-ATAATATCCAGGCAGTGTCCCGTGAATAAAACGGA-GTAAAAGTGGTAATGGTGTAGGCTGGAGCTGCTTC-3′ and ppk-right 5′-GAGGGGA-TTTATCGTGTATTGGCATAGGGTTATTCAGGTTGTTCGAGTGACATATGAATATCCTCCTTAG-3′ for amplifying the Km^R^ cartridge from plasmid pKD4.^80^ The chromosomal *ΔychFΔppx* double mutant was constructed by using *ΔychF* pBad24(Cm^R^)-YchF, pKD46 as parent strain and the oligonucleotides ppx-left 5′-GGCGATTTATGACTACATCAAA-TCACTCGAACAACCTGAATAACCCTATGGTGTAGGCTGGAGCTGCTTC-3′ and ppx-right 5′-GCCGACATTTCTCGTCGGCCCGCAAAGTATTAAGCGGCATTTCTGGTG-TCATATGAATATCCTCTTAG-3′ for amplifying the Km^R^ cartridge from plasmid pKD4.^80^ The *ychF-rpoS* double knock-out was generated using the *ΔychF* strain containing the plasmid pKD46 as parent strain and the oligonucleotides rpoS_left 5′-TGAGACTGGCCTTTCTGACAGATGCTTACTTACTCGCGGAACAGC-GCTTCCATA TGAATATCCTCCTTAG-3′ and rpoS_right 5′-CTTTTGCTTGAATGTTCCGTCAA-GGGATCACGGGTAGGAGCCACCTTATGGTGTAGGCTGGAGCTGCTTC-3'. Correct recombination and deletion was validated by PCR, sequencing and western-blotting. Plasmid pKD46 contains a temperature-sensitive origin and was eliminated from the double knock-outs by several passages at 37°C. Plasmid pBad24(Cm^R^)-YchF was constructed by replacing the ampicillin cartridge of pBad24-YchF^39^ with a Cm^R^ cartridge. Expression of *ychF* from pBad24 plasmids was induced by the addition of 0.0002% arabinose. For expression of *E. coli* PPX, the GST-Tag of plasmid pET22a-EcPPX^114^ was replaced with an N-terminal His-Tag. The plasmids pRS1-PPK and pRS1-PPX were constructed by amplifying the corresponding genome sequences from the *E. coli* BW5113 chromosome using the following oligonucleotides: F-RS1-PPK 5′-AAGAAGGAGATATACCCCATGGGTCAGGAA-AAGCTATACATG-3'; R-RS1-PPK 5′-GTCGACGAGCTCGCGGCCGCTTATTCAGGT-TGTTCGAGTGATTTG-3'; F-RS1-PPX 5′-AAGAAGGAGATATACCCCATGCCA-ATACACGATAAATCCCCTC-3′, R-RS1-PPX 5′-GTCGACGAGCTCGCGGCCGCTTA-AGCGGCGATTTCTGGTGTACTTTC-3'. The obtained PCR fragments were assembled in the plasmid pRS1^115^ for T7-dependent expression using the NEB Gibson Assembly Kit (New England Biolabs, USA).

#### Construction of *rpoS* translational fusions

The *rpoS-mcherry* fusion constructs were designed on a pBad24-backbone and contained either the mCherry coding sequence or the mCherry coding sequence preceded by the 606 nucleotide long 5′-UTR of rpoS; both constructs were generated by BioCat (Heidelberg, Germany). The native ribosome-binding site of pBad-5ÙTR-mCherry was removed by using the following primers pBAD_KO-RBSfw 5′-GAATTAACCATGGGCTGCTGTTGC-3′ and pBAD_KO-RBSrev 5′- TTAGCCCAAAAAACGGGTATGGAGAAA-3'. For mutating the 5′-UTR of RpoS in plasmid pBad-5UTR∗-mCherry, the following primers were used 5′UTR_mutaforw 5′-ATGGTGAGCAAGGGCGAGGAGGATAACATGGCC-3′ and 5′UTR_muta_rev 5′- ACCGTGGCTCCTACGGGTGATCCCTTGACGGAAC-3'.

All PCR reactions were performed with the NEB Q5 HiFi DNA-polymerase (New England Biolabs, USA) and PCR products were purified via the QIAGEN PCR purification kit (Qiagen, Hilden Germany).

#### RNA isolation and RT-PCR

For isolation of total RNA from 1 × 10^8^ cells, the QIAGEN RNeasy mini Kit was used. 1 × 10^8^ cells from the bacterial cultures were centrifuged at 5.000 rpm, 4°C for 10 min at 4°C. After removing the supernatant, the cells were resuspended in 700 μL RLT-buffer +1/100 β-mercaptoethanol. Heat pre-treated (180°C, overnight) and acid-washed glass beads (450–600 μm diameter) were added up to the 1 mL and the cells were shaken in the cell disruptor genius for 5 min. Afterward the 1.5 mL tube bottom was perforated with a hot cannula and the tube was placed on top of another 1.5 mL tube. The liquid was separated from the beads by centrifugation for 1 min at maximum 1.000 rpm at room temperature. In the following step one volume of 70% EtOH was added to the solution and the sample was loaded onto the supplied spin column and samples were processed as described in the QIAGEN RNeasy Mini Kit Handbook. The total RNA was eluted by adding 12 μL water to the column, followed by incubation at room temperature for 5 min and spinning at 13.000 rpm, 4°C for 1 min. The yield could be improved by reloading the eluate to the same column and repeating the elution steps. The isolated total RNA was afterward quantified using a spectrophotometer at 240 nm and the quality of the RNA preparation was tested by agarose electrophoresis. The total RNA was stored at −80°C.

For RT-PCR, the RNA was treated with DNase I (Sigma-Aldrich, Darmstadt, Germany) for 15 min at room temperature and DNase I was then inactivated by incubation at 70°C for 10 min. The reverse transcription reaction was performed in the presence of random hexameric primers, the Ribolock RNAse inhibitor (Thermo Fisher Scientific, Darmstadt, Germany) and the RevertAid reverse transcriptase (Thermo Fisher Scientific) for 60 min at 42°C. For the subsequent PCR and qPCR, the following primers were used RpoS-qPCRfw 5′-CGGAAGAGATCGCAGAGCAA-3′, RpoS-qPCRrev 5′- TGTCCAGCAACGCTTTTTCG-3'; HcaT-qPCRfw 5′-CCAACCACGCAGAC-CAACC-3′, HcaT-qPCRrev 5′-GCTGCTC-GGCTTTCTCATC-3'; KatE-qPCRfw 5′-CGTT-GGCAATAACACGCCAA-3′, KatE-qPCRrev 5′-CTTGTGGAATTGCCCAGTGGC-3'; OsmY-qPCRfw 5′-CGGTTTCGT-TGAAAGCCAGG-3`, OsmY-qPCRrev 5′-ACCGAGCC-TTCTTTAGCGTC-3′, PPK-fw 5′-ATGAATATTGTGGTGCTTATTTCCGGCAAC-3′, PPK-rev 5′-TTAAGCGGCGATTTCT-GGTGTACTT-3'; PPX-fw 5′-ATGGGTCAGGA-AAAGCTATACATCGAAAA-3`, PPX-rev 5'-ATGAAACTGAATGCAACTTATATAAA-AATACGTGATAAATGGTGG-3'. To ensure the quality of the analyses, RNA samples treated the same way but without reverse transcriptase were used as control samples in the qRT-PCR (-RT-samples). For quantification of the qRT-PCR, the threshold cycles (C_T_) were determined. The $2^{-C_{T}}$ values of the –RT-samples were subtracted from the $2^{-C_{T}}$ of the cDNA samples to create a new, background reduced C_T_ for each sample. Afterward, the C_T_ value for the gene of interest was subtracted from the C_T_ value of the housekeeping gene *hcat*. The fold change of expression of the target genes was calculated in regard to the expression in the wt cells in an analogous manner as described for the $2^{-\Delta \Delta C_{T}}$ method.^116^

#### SDS-polyacrylamide gel electrophoresis (SDS-PAGE) and western blot analyses

Samples were denatured at 56°C or 95°C for 10 min in Laemmli loading buffer (278 mM Tris-HCl, pH 6.8, 44.4% glycerol, 4.4% SDS, 0.02% bromophenol blue) containing fresh DTT at a final concentration of 25 mM. Samples were separated routinely on 12% SDS-PAGE gels. For the immune detection of proteins in whole cells, routinely 1 × 10^8^ cells were precipitated with 5% trichloroacetic acid (TCA, final concentration) and incubated for 30 min on ice. The sample was subsequently centrifuged at 30.000 × g and 4°C for 15 min, the pellet was resuspended in 20 μL of SDS loading buffer and incubated for 10 min at 56°C before loading on SDS-PAGE. The SDS-PAGE-separated proteins were then transferred onto nitrocellulose membranes (GE Healthcare) and the membranes were blocked with 5% milk powder in T-TBS buffer for at least 1 h before the addition of the primary antibodies.

α-YchF antibodies were raised in rabbits against the peptide VNEDGFENNPYLDQC.^27^ Antibodies against YidC and FtsY were raised against the purified proteins and have been reported before.^109^^,^^110^ Monoclonal antibodies against RpoS (clone 1RS1) were obtained from Biolegend (Amsterdam, Netherlands). Antibodies against PPK and PPX were raised in rabbits by Genscript (Rijswijk, Netherlands) against the following peptides IIISEEQGSNSHSRC and VEHTQPEKGRKLVIC, respectively. A horseradish peroxidase-coupled secondary antibody from Caltag Laboratories (Burlingam, CA, USA) was used for detection; blots were incubated for 1 min with home-made ECL reagent and signals were detected by a CCD camera. Western blot samples were analyzed by using *ImageQuant* (GE Healthcare) or the *ImageJ/Fiji* plug-in software (NIH, Bethesda, USA). All experiments were performed at least twice as independent biological replicates and representative gels/blots/images are shown. When data were quantified, at least three independent biological replicates with several technical replicates were performed and the signal intensity observed for wild type cells or cell extracts was set to 100%.

#### Protein purification

Protein purification of C-terminal His_6_-tagged YchF followed previously published protocols using *E. coli* BL21 containing the plasmid pBad24-YchF as expression host.^41^ N-terminal His_6_-tagged PPX was purified from BL21(DE3) pET22b-PPX after induction with 1 mM IPTG and growth for 3 h at 37°C. Cells were then harvested and resuspended in HKM buffer (25 mM HEPES (4-(2-hydroxyethyl)-1-piperazineethanesulfonic acid), pH 7.1, 200 mM KCl_2_, 10 mM MgCl_2_, 5% glycerol), containing the Complete protease inhibitor mix (Roche, Penzberg, Germany) and phenylmethylsulfonyl-fluorid (PMSF, 1 mM final concentration). Cells were lysed three times by a French pressure cell and cell debris were removed by centrifugation (30 min, 15.500 rpm, Lynx 6000 centrifuge with the F21 rotor, Thermo-Fisher Scientific). The supernatant was mixed with HKM-equilibrated Talon beads (Merck) and incubated for 1 h at 4°C on a rotary wheel. The material was then washed three time with HKM-buffer containing 5 mM imidazole, before elution with 5 mL HKM buffer containing 200 mM imidazole. The eluted material was then buffer exchanged against HKM buffer +10% glycerol using PD-10 columns. The material was analyzed by SDS-PAGE and protein concentration was determined by the BCA-assay (Thermo-Fisher Scientific). PPX was either directly used or stored at −20°C.

#### Polyphosphate determination

Polyphosphate determination in whole cells basically followed published protocols.^67^ Cells were grown overnight in LB medium, transferred to fresh LB-medium and grown up to an optical density of 0.6–0.7. Cells were then washed in minimal MOPS medium,^74^ containing 0.13 mM K_2_HPO_4_ and 0.2% glucose, and incubated further in minimal MOPS medium for 2 h at 37°C. 2 × 10^8^ cells were then incubated with 10 μM DAPI (4′,6-diamidino-2-phenylindole) in 100 mM Tris-HCl, pH 7.5 for 5 min at 37°C. Fluorescence was determined after excitation at 414 nm and emission at 550 nm with the Tecan Spark plate reader (QuadX monochromator; Xenon flash lamp; 20 nm bandwidth for excitation and emission). As internal control, the fluorescence of DAPI-stained DNA was also measured at an excitation of 358 nm and an emission of 461 nm.

For polyphosphate determination in cell extracts, *E. coli* cells were resuspended in cell lysis buffer (50 mM sodium acetate (pH 8.0), 10 mM EDTA, 0.4% SDS, 60 μg proteinase K) and incubated at 37°C for 20 min with continuous shaking. Subsequently, 450 μL of Tris-bufferd phenol (pH 8.0) and 450 μL chloroform were added and samples were mixed 3 times by vortexing for 5 s. Samples were centrifuged in a pre-cooled centrifuge at 18.000xg for 20 min at 4°C. 450 μL of the polyphosphate containing aqueous upper phase were transferred into a 1.5 mL Eppendorf tube containing 525 μL cold chloroform, mixed and centrifuged again at 18.000xg for 20 min at 4°C. The upper phase was transferred into a fresh 1.5 mL tube and 1 μL RNAse A (10 mg/mL) and 2 μL DNAse I (10 mg/mL) were added followed by incubation for 20 min at 37°C. The samples were precipitated overnight at −20°C with 40 μL 3 M sodium acetate (pH 5.3) and 1 mL 100% ethanol and then centrifuged in a pre-cooled centrifuge at 18.000xg for 20 min at 4°C. The supernatant was discarded and the pellet was washed with cold 70% ethanol, before drying the pellet at room temperature for 15 min. The extracted polyP was dissolved in reaction buffer (40 mM Tris-acetate, pH 8,0 10 mM Mg-acetate, 30 mM NH_4_-acetate, 0.2 mM EDTA) and 42 μL of this solution were incubated with purified *E. coli* PPX (final concentration 3 μM) for 1 h at 37°C in a final volume of 50 μL. The reaction was stopped by a 10 min incubation at 65°C.

The released phosphate was determined by the Malachite green assay kit (Sigma Aldrich, Darmstadt, Germany). In brief, the PPX reaction mix was diluted 1:5 or 1:10 with phosphate-free water and 80 μL of these dilutions were added to 20 μL of the Malachite green working solution in a micro-titer plate. Samples were carefully mixed and incubated for 30 min at room temperature before absorbance was measured at 620 nm. The obtained phosphate concentrations were then normalized to the protein content, which was determined via the BCA assay kit (Thermo-Fisher Scientific). Alternatively, the Phosfinity ChainQuant Kit (Aminoverse B.V., Netherlands) was used, which determines polyP levels via yeast PPX.^117^

#### Exopolyphosphatase assay

The PPX activity *in vitro* was determined basically as described above. Purified *E. coli* PPX (3 μM) was incubated with saturating concentrations of purified polyP_100_ or polyP_700_ (1 mM final concentration) (Kerafast Inc, Boston, USA) at 30°C. Samples were taken at different time points and the released phosphate was determined via the malachite green assay as described above. When indicated, the assay was performed in the presence of different YchF concentrations and 2 mM ATP.

#### Lipidomic analyses of inner membrane vesicles

Lipid composition was determined in inner membrane vesicles (INVs) of the indicated strains. Cells were grown on INV medium (10 g/L tryptone; 10 g/L yeast extract; 166 mM K_2_HPO_4_, 41 mM KH_2_PO_4_, 1% Glucose) up to OD_600_ of 1.5, harvested and washed twice in buffer A (50 mM TeaOAc, pH 7.5; 250 mM sucrose, 1 mM EDTA, 1 mM DTT). Cells were resuspended in 1 mL buffer A/g cell weight and lysed in a cooled French pressure cell. The lysate was then centrifuged at 150.000 g for 2 h in Beckmann Ti50.2 rotor and the pellet containing the membranes were resuspended in buffer A. These crude membranes were loaded on a discontinuous sucrose gradient (0.77 M; 1.44 M; 2.02 M sucrose in buffer A + 0.5 mM PMSF) and centrifuged in Sorvall SureSpin106.4 swing-out rotor at 25.000 rpm for 17 h. After centrifugation, the inner membrane phase was collected, diluted with 50 mM TeaOAc, pH 7.5 and centrifuged for 2 h at 150.000 g in a Beckmann TI50.2 rotor. The pellet was resuspended in buffer A and samples were stored in small aliquots at −80°C.

#### Lipid extraction for mass spectrometry lipidomics

Mass spectrometry-based lipid analysis was performed by Lipotype GmbH (Dresden, Germany) as described.^118^ Lipids were extracted using a two-step chloroform/methanol procedure.^119^ Samples were spiked with internal lipid standard mixture containing: cardiolipin 14:0/14:0/14:0/14:0 (CL), ceramide 18:1; 2/17:0 (Cer), diacylglycerol 17:0/17:0 (DAG), hexosylceramide 18:1; 2/12:0 (HexCer), lyso-phosphatidate 17:0 (LPA), lyso-phosphatidylcholine 12:0 (LPC), lyso-phosphatidylethanolamine 17:1 (LPE), lyso-phosphatidylglycerol 17:1 (LPG), lyso-phosphatidylinositol 17:1 (LPI), lyso-phosphatidylserine 17:1 (LPS), phosphatidate 17:0/17:0 (PA), phosphatidylcholine 17:0/17:0 (PC), phosphatidylethanolamine 17:0/17:0 (PE), phosphatidylglycerol 17:0/17:0 (PG), phosphatidylinositol 16:0/16:0 (PI), phosphatidylserine 17:0/17:0 (PS), cholesterolester 20:0 (CE), sphingomyelin 18:1; 2/12:0; 0 (SM), triacylglycerol 17:0/17:0/17:0 (TAG). After extraction, the organic phase was transferred to an infusion plate and dried in a speed vacuum concentrator. 1st step dry extract was resuspended in 7.5 mM ammonium acetate in chloroform/methanol/propanol (1:2:4, V:V:V) and 2nd step dry extract in 33% ethanol solution of methylamine in chloroform/methanol (0.003:5:1; V:V:V). All liquid handling steps were performed using Hamilton Robotics STARlet robotic platform with the Anti Droplet Control feature for organic solvents pipetting.

#### MS data acquisition

Samples were analyzed by direct infusion on a QExactive mass spectrometer (Thermo Scientific) equipped with a TriVersa NanoMate ion source (Advion Biosciences). Samples were analyzed in both positive and negative ion modes with a resolution of Rm/z = 200 = 280000 for MS and Rm/z = 200 = 17500 for MSMS experiments, in a single acquisition. MSMS was triggered by an inclusion list encompassing corresponding MS mass ranges scanned in 1 Da increments.^120^ Both MS and MSMS data were combined to monitor CE, DAG and TAG ions as ammonium adducts; PC, PC O-, as acetate adducts; and CL, PA, PE, PE O-, PG, PI and PS as deprotonated anions. MS only was used to monitor LPA, LPE, LPE O-, LPI and LPS as deprotonated anions; Cer, HexCer, SM, LPC and LPC O- as acetate adducts.

#### Data analysis and post-processing

Data were analyzed with in-house developed lipid identification software based on LipidXplorer.^121^ Data post-processing and normalization were performed using an in-house developed data management system. Only lipid identifications with a signal-to-noise ratio >5, and a signal intensity 5-fold higher than in corresponding blank samples were considered for further data analysis.

#### Fluoresence measurements of *rpoS*-mCherry fusions

The plasmids pBAD_mChe, pBAD-5UTR-mCherry and pBad5UTR∗-mCherry were transformed into *E. coli* strains BW25113 (wt) and JW1194 (Δ*ychF*, Km^S^) and cells were grown on LB medium at 37°C with shaking (180 rpm) up to an optical density of 1.0. Cells were then harvested, washed with PBS-buffer and their optical density was again adjusted to 1.0. 100 μL of each culture was then transferred to black 96 well plate (Greiner) and fluorescence was measured in a TECAN Spark plate reader after excitation at 576 nm and an emission of 610 nm with gain 97.

### Quantification and statistical analysis

Western blot and autoradiography samples were analyzed by using the *ImageQuant* (GE Healthcare) or the *ImageJ/Fiji* plug-in software (NIH, Bethesda, USA). All experiments were performed at least twice as independent biological replicates and representative gels/blots/images are shown. When data were quantified, at least three independent biological replicates with several technical replicates were performed. Mean values and standard deviations were determined by using either Excel (Microsoft Corp.) or GraphPad Prism (GraphPad Prism Corp. San Diego). For statistical analyses, a Student unpaired two-way t-test was performed.

## References

[1] Costa-Mattioli M., Walter P. (2020). The integrated stress response: From mechanism to disease. Science.

[2] Pan T. (2013). Adaptive translation as a mechanism of stress response and adaptation. Annu. Rev. Genet..

[3] Landwehr V., Milanov M., Hong J., Koch H.G. (2021). The Role of the Universally Conserved ATPase YchF/Ola1 in Translation Regulation during Cellular Stress. Microorganisms.

[4] Njenga R., Boele J., Öztürk Y., Koch H.G. (2023). Coping with stress: how bacteria fine-tune protein synthesis and protein transport. J. Biol. Chem..

[5] Rother M., Teixeira da Costa A.R., Zietlow R., Meyer T.F., Rudel T. (2019). Modulation of Host Cell Metabolism by Chlamydia trachomatis. Microbiol. Spectr..

[6] Cornforth D.M., Foster K.R. (2013). Competition sensing: the social side of bacterial stress responses. Nat. Rev. Microbiol..

[7] Oliveira N.M., Wheeler J.H.R., Deroy C., Booth S.C., Walsh E.J., Durham W.M., Foster K.R. (2022). Suicidal chemotaxis in bacteria. Nat. Commun..

[8] Cheng-Guang H., Gualerzi C.O. (2020). The Ribosome as a Switchboard for Bacterial Stress Response. Front. Microbiol..

[9] Gottesman S. (2019). Trouble is coming: Signaling pathways that regulate general stress responses in bacteria. J. Biol. Chem..

[10] Guo M.S., Gross C.A. (2014). Stress-induced remodeling of the bacterial proteome. Curr. Biol..

[11] Schellhorn H.E. (2020). Function, Evolution, and Composition of the RpoS Regulon in Escherichia coli. Front. Microbiol..

[12] Abril A.G., Rama J.L.R., Sánchez-Pérez A., Villa T.G. (2020). Prokaryotic sigma factors and their transcriptional counterparts in Archaea and Eukarya. Appl. Microbiol. Biotechnol..

[13] Girard M.E., Gopalkrishnan S., Grace E.D., Halliday J.A., Gourse R.L., Herman C. (2018). DksA and ppGpp Regulate the σ(S) Stress Response by Activating Promoters for the Small RNA DsrA and the Anti-Adapter Protein IraP. J. Bacteriol..

[14] Gray M.J. (2019). Inorganic Polyphosphate Accumulation in Escherichia coli Is Regulated by DksA but Not by (p)ppGpp. J. Bacteriol..

[15] Altuvia S., Weinstein-Fischer D., Zhang A., Postow L., Storz G. (1997). A small, stable RNA induced by oxidative stress: role as a pleiotropic regulator and antimutator. Cell.

[16] Gottesman S., Storz G. (2011). Bacterial small RNA regulators: versatile roles and rapidly evolving variations. Cold Spring Harb. Perspect. Biol..

[17] Jorgensen M.G., Thomason M.K., Havelund J., Valentin-Hansen P., Storz G. (2013). Dual function of the McaS small RNA in controlling biofilm formation. Genes Dev..

[18] Gottesman S., Storz G., Rosenow C., Majdalani N., Repoila F., Wassarman K.M. (2001). Small RNA regulators of translation: mechanisms of action and approaches for identifying new small RNAs. Cold Spring Harb. Symp. Quant. Biol..

[19] Leiva L.E., Zegarra V., Bange G., Ibba M. (2023). At the Crossroad of Nucleotide Dynamics and Protein Synthesis in Bacteria. Microbiol. Mol. Biol. Rev..

[20] Zegarra V., Bedrunka P., Bange G., Czech L. (2023). How to save a bacterial ribosome in times of stress. Semin. Cell Dev. Biol..

[21] Czech L., Mais C.N., Kratzat H., Sarmah P., Giammarinaro P., Freibert S.A., Esser H.F., Musial J., Berninghausen O., Steinchen W. (2022). Inhibition of SRP-dependent protein secretion by the bacterial alarmone (p)ppGpp. Nat. Commun..

[22] Sarmah P., Shang W., Origi A., Licheva M., Kraft C., Ulbrich M., Lichtenberg E., Wilde A., Koch H.G. (2023). mRNA targeting eliminates the need for the signal recognition particle during membrane protein insertion in bacteria. Cell Rep..

[23] Barik S. (2023). Protein-Ligand Interactions in Scarcity: The Stringent Response from Bacteria to Metazoa, and the Unanswered Questions. Int. J. Mol. Sci..

[24] Balasingam N., Brandon H.E., Ross J.A., Wieden H.J., Thakor N. (2020). Cellular roles of the human Obg-like ATPase 1 (hOLA1) and its YchF homologs. Biochem. Cell. Biol..

[25] Koller-Eichhorn R., Marquardt T., Gail R., Wittinghofer A., Kostrewa D., Kutay U., Kambach C. (2007). Human OLA1 defines an ATPase subfamily in the Obg family of GTP-binding proteins. J. Biol. Chem..

[26] Liu J., Miao X., Xiao B., Huang J., Tao X., Zhang J., Zhao H., Pan Y., Wang H., Gao G., Xiao G.G. (2020). Obg-Like ATPase 1 Enhances Chemoresistance of Breast Cancer via Activation of TGF-β/Smad Axis Cascades. Front. Pharmacol..

[27] Wenk M., Ba Q., Erichsen V., MacInnes K., Wiese H., Warscheid B., Koch H.G. (2012). A universally conserved ATPase regulates the oxidative stress response in Escherichia coli. J. Biol. Chem..

[28] Zhang J., Rubio V., Lieberman M.W., Shi Z.Z. (2009). OLA1, an Obg-like ATPase, suppresses antioxidant response via nontranscriptional mechanisms. Proc. Natl. Acad. Sci. USA.

[29] Verstraeten N., Fauvart M., Versées W., Michiels J. (2011). The universally conserved prokaryotic GTPases. Microbiol. Mol. Biol. Rev..

[30] Chen H., Song R., Wang G., Ding Z., Yang C., Zhang J., Zeng Z., Rubio V., Wang L., Zu N. (2015). OLA1 regulates protein synthesis and integrated stress response by inhibiting eIF2 ternary complex formation. Sci. Rep..

[31] Dannenmaier S., Desroches Altamirano C., Schüler L., Zhang Y., Hummel J., Milanov M., Oeljeklaus S., Koch H.G., Rospert S., Alberti S., Warscheid B. (2021). Quantitative proteomics identifies the universally conserved ATPase Ola1p as a positive regulator of heat shock response in Saccharomyces cerevisiae. J. Biol. Chem..

[32] Mao R.F., Rubio V., Chen H., Bai L., Mansour O.C., Shi Z.Z. (2013). OLA1 protects cells in heat shock by stabilizing HSP70. Cell Death Dis..

[33] Zhang J.W., Rubio V., Zheng S., Shi Z.Z. (2009). Knockdown of OLA1, a regulator of oxidative stress response, inhibits motility and invasion of breast cancer cells. J. Zhejiang Univ. Sci. B.

[34] Cheung M.Y., Li M.W., Yung Y.L., Wen C.Q., Lam H.M. (2013). The unconventional P-loop NTPase OsYchF1 and its regulator OsGAP1 play opposite roles in salinity stress tolerance. Plant Cell Environ..

[35] Cheung M.Y., Li X., Miao R., Fong Y.H., Li K.P., Yung Y.L., Yu M.H., Wong K.B., Chen Z., Lam H.M. (2016). ATP binding by the P-loop NTPase OsYchF1 (an unconventional G protein) contributes to biotic but not abiotic stress responses. Proc. Natl. Acad. Sci. USA.

[36] Sidlowski P., Czerwinski A., Liu Y., Liu P., Teng R.J., Kumar S., Wells C., Pritchard K., Konduri G.G., Afolayan A.J. (2023). OLA1 Phosphorylation Governs the Mitochondrial Bioenergetic Function of Pulmonary Vascular Cells. Am. J. Respir. Cell Mol. Biol..

[37] Liu J., Yang Q., Xiao K.C., Dobleman T., Hu S., Xiao G.G. (2020). Obg-like ATPase 1 inhibited oral carcinoma cell metastasis through TGFβ/SMAD2 axis in vitro. BMC Mol. Cell Biol..

[38] Sun H., Luo X., Montalbano J., Jin W., Shi J., Sheikh M.S., Huang Y. (2010). DOC45, a novel DNA damage-regulated nucleocytoplasmic ATPase that is overexpressed in multiple human malignancies. Mol. Cancer Res..

[39] Hannemann L., Suppanz I., Ba Q., MacInnes K., Drepper F., Warscheid B., Koch H.G. (2016). Redox Activation of the Universally Conserved ATPase YchF by Thioredoxin 1. Antioxid. Redox Signal..

[40] Becker M., Gzyl K.E., Altamirano A.M., Vuong A., Urban K., Wieden H.J. (2012). The 70S ribosome modulates the ATPase activity of Escherichia coli YchF. RNA Biol..

[41] Landwehr V., Milanov M., Angebauer L., Hong J., Jüngert G., Hiersemenzel A., Siebler A., Schmit F., Öztürk Y., Dannenmaier S. (2021). The Universally Conserved ATPase YchF Regulates Translation of Leaderless mRNA in Response to Stress Conditions. Front. Mol. Biosci..

[42] Samanfar B., Tan L.H., Shostak K., Chalabian F., Wu Z., Alamgir M., Sunba N., Burnside D., Omidi K., Hooshyar M. (2014). A global investigation of gene deletion strains that affect premature stop codon bypass in yeast, Saccharomyces cerevisiae. Mol. Biosyst..

[43] Tomar S.K., Kumar P., Prakash B. (2011). Deciphering the catalytic machinery in a universally conserved ribosome binding ATPase YchF. Biochem. Biophys. Res. Commun..

[44] Fang Z., Li X., Yoshino Y., Suzuki M., Qi H., Murooka H., Katakai R., Shirota M., Mai Pham T.A., Matsuzawa A. (2023). Aurora A polyubiquitinates the BRCA1-interacting protein OLA1 to promote centrosome maturation. Cell Rep..

[45] Hirsch M., Elliott T. (2005). Stationary-phase regulation of RpoS translation in Escherichia coli. J. Bacteriol..

[46] Kolodkin-Gal I., Engelberg-Kulka H. (2009). The stationary-phase sigma factor sigma(S) is responsible for the resistance of Escherichia coli stationary-phase cells to mazEF-mediated cell death. J. Bacteriol..

[47] Wong G.T., Bonocora R.P., Schep A.N., Beeler S.M., Lee Fong A.J., Shull L.M., Batachari L.E., Dillon M., Evans C., Becker C.J. (2017). Genome-Wide Transcriptional Response to Varying RpoS Levels in Escherichia coli K-12. J. Bacteriol..

[48] Jishage M., Kvint K., Shingler V., Nyström T. (2002). Regulation of sigma factor competition by the alarmone ppGpp. Genes Dev..

[49] Zhou K., Zhou L., Lim Q.'E., Zou R., Stephanopoulos G., Too H.P. (2011). Novel reference genes for quantifying transcriptional responses of Escherichia coli to protein overexpression by quantitative PCR. BMC Mol. Biol..

[50] Yim H.H., Villarejo M. (1992). osmY, a new hyperosmotically inducible gene, encodes a periplasmic protein in Escherichia coli. J. Bacteriol..

[51] Tanaka K., Handel K., Loewen P.C., Takahashi H. (1997). Identification and analysis of the rpoS-dependent promoter of katE, encoding catalase HPII in Escherichia coli. Biochim. Biophys. Acta.

[52] Hiraoka S., Matsuzaki H., Shibuya I. (1993). Active increase in cardiolipin synthesis in the stationary growth phase and its physiological significance in Escherichia coli. FEBS Lett..

[53] Tan B.K., Bogdanov M., Zhao J., Dowhan W., Raetz C.R.H., Guan Z. (2012). Discovery of a cardiolipin synthase utilizing phosphatidylethanolamine and phosphatidylglycerol as substrates. Proc. Natl. Acad. Sci. USA.

[54] Ryabichko S., Ferreira V.d.M., Vitrac H., Kiyamova R., Dowhan W., Bogdanov M. (2020). Cardiolipin is required in vivo for the stability of bacterial translocon and optimal membrane protein translocation and insertion. Sci. Rep..

[55] Romantsov T., Helbig S., Culham D.E., Gill C., Stalker L., Wood J.M. (2007). Cardiolipin promotes polar localization of osmosensory transporter ProP in Escherichia coli. Mol. Microbiol..

[56] Traxler M.F., Summers S.M., Nguyen H.T., Zacharia V.M., Hightower G.A., Smith J.T., Conway T. (2008). The global, ppGpp-mediated stringent response to amino acid starvation in Escherichia coli. Mol. Microbiol..

[57] Kim M., Kim K.S. (2017). Stress-responsively modulated ymdAB-clsC operon plays a role in biofilm formation and apramycin susceptibility in Escherichia coli. FEMS Microbiol. Lett..

[58] Becker G., Klauck E., Hengge-Aronis R. (1999). Regulation of RpoS proteolysis in Escherichia coli: the response regulator RssB is a recognition factor that interacts with the turnover element in RpoS. Proc. Natl. Acad. Sci. USA.

[59] Schweder T., Lee K.H., Lomovskaya O., Matin A. (1996). Regulation of Escherichia coli starvation sigma factor (sigma s) by ClpXP protease. J. Bacteriol..

[60] Steinberg R., Knupffer L., Origi A., Asti R., Koch H.G. (2018). Co-translational protein targeting in bacteria. FEMS Microbiol. Lett..

[61] Vecerek B., Beich-Frandsen M., Resch A., Bläsi U. (2010). Translational activation of rpoS mRNA by the non-coding RNA DsrA and Hfq does not require ribosome binding. Nucleic Acids Res..

[62] Xie L., Jakob U. (2019). Inorganic polyphosphate, a multifunctional polyanionic protein scaffold. J. Biol. Chem..

[63] Bowlin M.Q., Gray M.J. (2021). Inorganic polyphosphate in host and microbe biology. Trends Microbiol..

[64] Bentley-DeSousa A., Downey M. (2021). Vtc5 Is Localized to the Vacuole Membrane by the Conserved AP-3 Complex to Regulate Polyphosphate Synthesis in Budding Yeast. mBio.

[65] Loewen P.C., Hu B., Strutinsky J., Sparling R. (1998). Regulation in the rpoS regulon of Escherichia coli. Can. J. Microbiol..

[66] Shiba T., Tsutsumi K., Yano H., Ihara Y., Kameda A., Tanaka K., Takahashi H., Munekata M., Rao N.N., Kornberg A. (1997). Inorganic polyphosphate and the induction of rpoS expression. Proc. Natl. Acad. Sci. USA.

[67] Aschar-Sobbi R., Abramov A.Y., Diao C., Kargacin M.E., Kargacin G.J., French R.J., Pavlov E. (2008). High sensitivity, quantitative measurements of polyphosphate using a new DAPI-based approach. J. Fluoresc..

[68] Ahn K., Kornberg A. (1990). Polyphosphate kinase from Escherichia coli. Purification and demonstration of a phosphoenzyme intermediate. J. Biol. Chem..

[69] Rao N.N., Liu S., Kornberg A. (1998). Inorganic polyphosphate in Escherichia coli: the phosphate regulon and the stringent response. J. Bacteriol..

[70] Dahl J.U., Gray M.J., Bazopoulou D., Beaufay F., Lempart J., Koenigsknecht M.J., Wang Y., Baker J.R., Hasler W.L., Young V.B. (2017). The anti-inflammatory drug mesalamine targets bacterial polyphosphate accumulation. Nat. Microbiol..

[71] Omelon S., Georgiou J., Habraken W. (2016). A cautionary (spectral) tail: red-shifted fluorescence by DAPI-DAPI interactions. Biochem. Soc. Trans..

[72] Kolozsvari B., Parisi F., Saiardi A. (2014). Inositol phosphates induce DAPI fluorescence shift. Biochem. J..

[73] Akiyama M., Crooke E., Kornberg A. (1993). An exopolyphosphatase of Escherichia coli. The enzyme and its ppx gene in a polyphosphate operon. J. Biol. Chem..

[74] Neidhardt F.C., Bloch P.L., Smith D.F. (1974). Culture medium for enterobacteria. J. Bacteriol..

[75] Bowlin M.Q., Long A.R., Huffines J.T., Gray M.J. (2022). The role of nitrogen-responsive regulators in controlling inorganic polyphosphate synthesis in Escherichia coli. Microbiology (Read.).

[76] Ault-Riché D., Fraley C.D., Tzeng C.M., Kornberg A. (1998). Novel assay reveals multiple pathways regulating stress-induced accumulations of inorganic polyphosphate in Escherichia coli. J. Bacteriol..

[77] Li G.W., Burkhardt D., Gross C., Weissman J.S. (2014). Quantifying absolute protein synthesis rates reveals principles underlying allocation of cellular resources. Cell.

[78] Kuroda A., Murphy H., Cashel M., Kornberg A. (1997). Guanosine tetra- and pentaphosphate promote accumulation of inorganic polyphosphate in Escherichia coli. J. Biol. Chem..

[79] Steinchen W., Zegarra V., Bange G. (2020). (p)ppGpp: Magic Modulators of Bacterial Physiology and Metabolism. Front. Microbiol..

[80] Datsenko K.A., Wanner B.L. (2000). One-step inactivation of chromosomal genes in Escherichia coli K12 using PCR products. Proc. Natl. Acad. Sci. USA.

[81] Maciag A., Peano C., Pietrelli A., Egli T., De Bellis G., Landini P. (2011). In vitro transcription profiling of the σS subunit of bacterial RNA polymerase: re-definition of the σS regulon and identification of σS-specific promoter sequence elements. Nucleic Acids Res..

[82] Singh A., Xu Y.J. (2016). The Cell Killing Mechanisms of Hydroxyurea. Genes.

[83] Juul T., Malolepszy A., Dybkaer K., Kidmose R., Rasmussen J.T., Andersen G.R., Johnsen H.E., Jørgensen J.E., Andersen S.U. (2010). The in vivo toxicity of hydroxyurea depends on its direct target catalase. J. Biol. Chem..

[84] Grosjean H., Breton M., Sirand-Pugnet P., Tardy F., Thiaucourt F., Citti C., Barré A., Yoshizawa S., Fourmy D., de Crécy-Lagard V., Blanchard A. (2014). Predicting the minimal translation apparatus: lessons from the reductive evolution of mollicutes. PLoS Genet..

[85] Ding Z., Liu Y., Rubio V., He J., Minze L.J., Shi Z.Z. (2016). Ola1, a translational regulator of p21, maintains optimal cell proliferation necessary for developmental progression. Mol. Cell Biol..

[86] Schneider-Poetsch T., Ju J., Eyler D.E., Dang Y., Bhat S., Merrick W.C., Green R., Shen B., Liu J.O. (2010). Inhibition of eukaryotic translation elongation by cycloheximide and lactimidomycin. Nat. Chem. Biol..

[87] Vijayakumar S.R.V., Kirchhof M.G., Patten C.L., Schellhorn H.E. (2004). RpoS-regulated genes of Escherichia coli identified by random lacZ fusion mutagenesis. J. Bacteriol..

[88] Cunning C., Brown L., Elliott T. (1998). Promoter substitution and deletion analysis of upstream region required for rpoS translational regulation. J. Bacteriol..

[89] Teplyakov A., Obmolova G., Chu S.Y., Toedt J., Eisenstein E., Howard A.J., Gilliland G.L. (2003). Crystal structure of the YchF protein reveals binding sites for GTP and nucleic acid. J. Bacteriol..

[90] Brescia C.C., Kaw M.K., Sledjeski D.D. (2004). The DNA binding protein H-NS binds to and alters the stability of RNA in vitro and in vivo. J. Mol. Biol..

[91] Peterson C.N., Carabetta V.J., Chowdhury T., Silhavy T.J. (2006). LrhA regulates rpoS translation in response to the Rcs phosphorelay system in Escherichia coli. J. Bacteriol..

[92] Storz G., Tartaglia L.A., Ames B.N. (1990). Transcriptional regulator of oxidative stress-inducible genes: direct activation by oxidation. Science.

[93] Zheng M., Wang X., Templeton L.J., Smulski D.R., LaRossa R.A., Storz G. (2001). DNA microarray-mediated transcriptional profiling of the Escherichia coli response to hydrogen peroxide. J. Bacteriol..

[94] González-Flecha B., Demple B. (1997). Transcriptional regulation of the Escherichia coli oxyR gene as a function of cell growth. J. Bacteriol..

[95] Bondy-Chorney E., Abramchuk I., Nasser R., Holinier C., Denoncourt A., Baijal K., McCarthy L., Khacho M., Lavallée-Adam M., Downey M. (2020). A Broad Response to Intracellular Long-Chain Polyphosphate in Human Cells. Cell Rep..

[96] Gray M.J., Jakob U. (2015). Oxidative stress protection by polyphosphate--new roles for an old player. Curr. Opin. Microbiol..

[97] Wang L., Fraley C.D., Faridi J., Kornberg A., Roth R.A. (2003). Inorganic polyphosphate stimulates mammalian TOR, a kinase involved in the proliferation of mammary cancer cells. Proc. Natl. Acad. Sci. USA.

[98] Kus F., Smolenski R.T., Tomczyk M. (2022). Inorganic Polyphosphate-Regulator of Cellular Metabolism in Homeostasis and Disease. Biomedicines.

[99] Peng L., Jiang Q., Pan J.Y., Deng C., Yu J.Y., Wu X.M., Huang S.H., Deng X.Y. (2016). Involvement of polyphosphate kinase in virulence and stress tolerance of uropathogenic Proteus mirabilis. Med. Microbiol. Immunol..

[100] Denoncourt A., Downey M. (2021). Model systems for studying polyphosphate biology: a focus on microorganisms. Curr. Genet..

[101] Rudat A.K., Pokhrel A., Green T.J., Gray M.J. (2018). Mutations in Escherichia coli Polyphosphate Kinase That Lead to Dramatically Increased In Vivo Polyphosphate Levels. J. Bacteriol..

[102] Arredondo C., Cefaliello C., Dyrda A., Jury N., Martinez P., Díaz I., Amaro A., Tran H., Morales D., Pertusa M. (2022). Excessive release of inorganic polyphosphate by ALS/FTD astrocytes causes non-cell-autonomous toxicity to motoneurons. Neuron.

[103] Ruiz N., Silhavy T.J. (2003). Constitutive activation of the Escherichia coli Pho regulon upregulates rpoS translation in an Hfq-dependent fashion. J. Bacteriol..

[104] Jeyabal P.V.S., Rubio V., Chen H., Zhang J., Shi Z.Z. (2014). Regulation of cell-matrix adhesion by OLA1, the Obg-like ATPase 1. Biochem. Biophys. Res. Commun..

[105] Tembe V., Martino-Echarri E., Marzec K.A., Mok M.T.S., Brodie K.M., Mills K., Lei Y., DeFazio A., Rizos H., Kettle E. (2015). The BARD1 BRCT domain contributes to p53 binding, cytoplasmic and mitochondrial localization, and apoptotic function. Cell. Signal..

[106] Xu D., Song R., Wang G., Jeyabal P.V.S., Weiskoff A.M., Ding K., Shi Z.Z. (2016). Obg-like ATPase 1 regulates global protein serine/threonine phosphorylation in cancer cells by suppressing the GSK3β-inhibitor 2-PP1 positive feedback loop. Oncotarget.

[107] Takahashi M., Chiba N., Shimodaira H., Yoshino Y., Mori T., Sumii M., Nomizu T., Ishioka C. (2017). OLA1 gene sequencing in patients with BRCA1/2 mutation-negative suspected hereditary breast and ovarian cancer. Breast Cancer.

[108] Samper-Martín B., Sarrias A., Lázaro B., Pérez-Montero M., Rodríguez-Rodríguez R., Ribeiro M.P.C., Bañón A., Wolfgeher D., Jessen H.J., Alsina B. (2021). Polyphosphate degradation by Nudt3-Zn(2+) mediates oxidative stress response. Cell Rep..

[109] Koch H.G., Moser M., Schimz K.L., Muller M. (2002). The integration of YidC into the cytoplasmic membrane of Escherichia coli requires the signal recognition particle, SecA and SecYEG. J. Biol. Chem..

[110] Koch H.G., Hengelage T., Neumann-Haefelin C., MacFarlane J., Hoffschulte H.K., Schimz K.L., Mechler B., Müller M. (1999). In vitro studies with purified components reveal signal recognition particle (SRP) and SecA/SecB as constituents of two independent protein-targeting pathways of Escherichia coli. Mol. Biol. Cell.

[111] Hanahan D. (1983). Studies on transformation of Escherichia coli with plasmids. J. Mol. Biol..

[112] Miroux B., Walker J.E. (1996). Over-production of proteins in Escherichia coli: mutant hosts that allow synthesis of some membrane proteins and globular proteins at high levels. J. Mol. Biol..

[113] Baba T., Ara T., Hasegawa M., Takai Y., Okumura Y., Baba M., Datsenko K.A., Tomita M., Wanner B.L., Mori H. (2006). Construction of Escherichia coli K-12 in-frame, single-gene knockout mutants: the Keio collection. Mol. Syst. Biol..

[114] Azevedo C., Livermore T., Saiardi A. (2015). Protein polyphosphorylation of lysine residues by inorganic polyphosphate. Mol. Cell.

[115] Jauss B., Petriman N.A., Drepper F., Franz L., Sachelaru I., Welte T., Steinberg R., Warscheid B., Koch H.G. (2019). Noncompetitive binding of PpiD and YidC to the SecYEG translocon expands the global view on the SecYEG interactome in *Escherichia coli*. J. Biol. Chem..

[116] Cannon K.S., Or E., Clemons W.M., Shibata Y., Rapoport T.A. (2005). Disulfide bridge formation between SecY and a translocating polypeptide localizes the translocation pore to the center of SecY. J. Cell Biol..

[117] Bru S., Jiménez J., Canadell D., Ariño J., Clotet J. (2016). Improvement of biochemical methods of polyP quantification. Microb. Cell.

[118] Sampaio J.L., Gerl M.J., Klose C., Ejsing C.S., Beug H., Simons K., Shevchenko A. (2011). Membrane lipidome of an epithelial cell line. Proc. Natl. Acad. Sci. USA.

[119] Ejsing C.S., Sampaio J.L., Surendranath V., Duchoslav E., Ekroos K., Klemm R.W., Simons K., Shevchenko A. (2009). Global analysis of the yeast lipidome by quantitative shotgun mass spectrometry. Proc. Natl. Acad. Sci. USA.

[120] Surma M.A., Herzog R., Vasilj A., Klose C., Christinat N., Morin-Rivron D., Simons K., Masoodi M., Sampaio J.L. (2015). An automated shotgun lipidomics platform for high throughput, comprehensive, and quantitative analysis of blood plasma intact lipids. Eur. J. Lipid Sci. Technol..

[121] Herzog R., Schwudke D., Shevchenko A. (2013). LipidXplorer: Software for Quantitative Shotgun Lipidomics Compatible with Multiple Mass Spectrometry Platforms. Curr. Protoc. Bioinformatics.

