# Supplementary material for: Control of a chemical chaperone by a universally conserved ATPase
Source: iScience. 2024 Jun 8;27(7):110215. doi: 10.1016/j.isci.2024.110215 (PMC11237923; doi:10.1016/j.isci.2024.110215)
Supplement: Document S1. Figures S1 and S2 [file mmc1.pdf]

iScience, Volume *27*

## Supplemental information

# Control of a chemical chaperone by a universally conserved ATPase

**Hong Jiang, Martin Milanov, Gabriela Jüngert, Larissa Angebauer, Clara Flender, Eva Smudde, Fabian Gather, Tanja Vogel, Henning J. Jessen, and Hans-Georg Koch**

## Supplementary Figures

**Figure S1: Growth-phase dependent RpoS levels, related to Figure 1.** The *E. coli* strains were grown on LB medium up to the indicated OD and 1 x $10^8$ cells were precipitated with 5 % trichloroacetic acid (TCA) before SDS-PAGE and western blotting. The membrane was then cut and incubated with α-RpoS antibodies (upper panel) or α-YidC antibodies (lower panel). Please note that due to the limited number of gel wells, the ΔychF + pYchF sample at OD 2.1 is not shown.

**Figure S2: Hydroxyurea resistance of *E. coli* single knock-out strains, related to Figure 6.** The indicated strains were grown on LB medium to $OD_{600}$ of ~ 1.0 and pelleted. Cells were washed with PBS and serially diluted in PBS. Of each dilution, starting with $10^7$ cells, 10 µl cell suspensions were spotted onto LB plates and LB plates containing, 5 mM, 7.5 mM or 10 mM hydroxyurea. Cell growth was monitored after overnight incubation at 37° C.

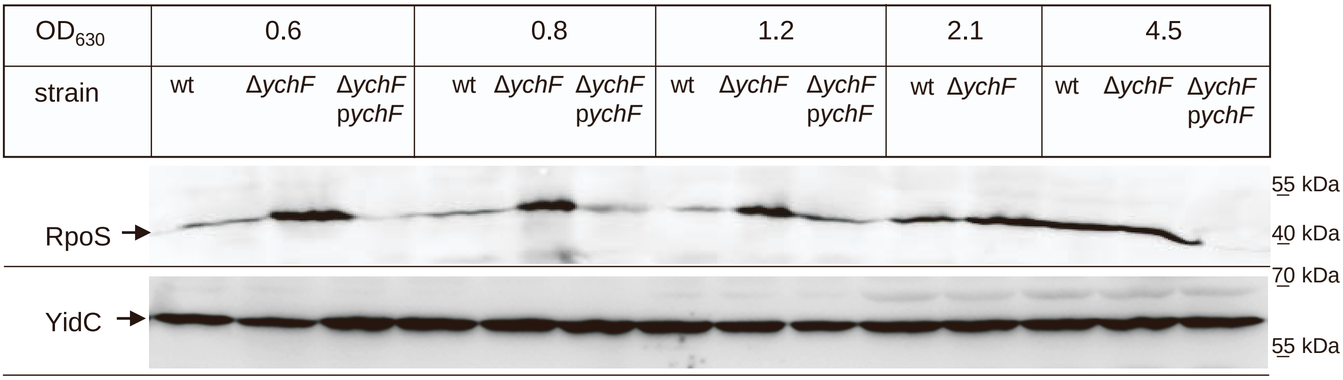


(Jiang et al., Supp. Fig. 1)

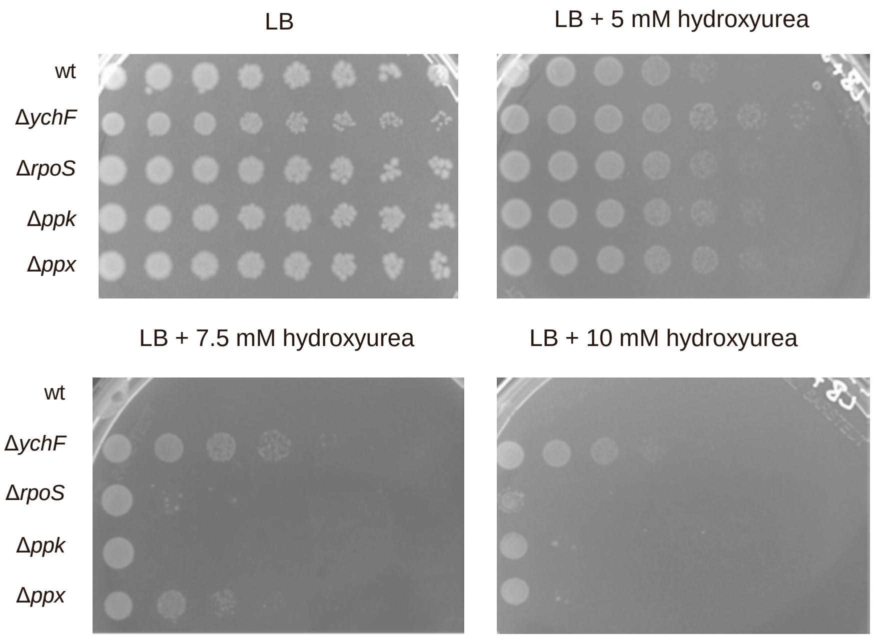


(Jiang et al., Supp. Fig. 2)
